# Unveiling the mechanisms of critical and high-value metals extraction from secondary resources *via* molten salt electrolysis: a review

**DOI:** 10.1039/d6ra08998d

**Published:** 2026-11-10

**Authors:** Shuaixing Wang, Ming Feng, Jieyang Sun, Binyao Zhang, Yanfen Li

**Affiliations:** a School of Materials Science and Engineering, Inner Mongolia University of Technology Hohhot 010051 P. R. China fengming@imut.edu.cn

## Abstract

With the rapid growth in demand for critical metals in industries such as energy storage and advanced materials, secondary resources are increasingly emerging as promising alternatives to primary raw materials. Traditional methods for extracting metals from secondary resources often face limitations in dealing with complex compositions and harsh processing conditions. Molten-salt electrolysis has shown considerable potential for the recovery of a wide range of metals from various secondary resources, owing to its advantages in high-temperature ionic environments, efficient mass transport, and direct electrochemical conversion. However, a systematic understanding of reaction pathways and mechanisms remains limited. Existing studies also lack comprehensive analyses of coupled electrochemical, structural, and interfacial processes governing metal extraction. Therefore, considering their potential as inexpensive and readily available sources of critical metals, this review evaluates various industrial and mineral processing wastes—including metal/alloy waste, coal ash, and other secondary resources. This review analyzes spectroscopic characterization, theoretical simulations, and reaction mechanisms to advance extraction technologies and address key challenges. The environmental implications of molten-salt electrolysis are also evaluated through life cycle assessment, providing further insight into its environmental performance and potential for large-scale application.

## Abstract 2

The increasing demand for critical metals in energy storage and advanced materials highlights the importance of secondary resource recovery, with molten salt electrolysis offering a promising approach.

## Graphical abstract

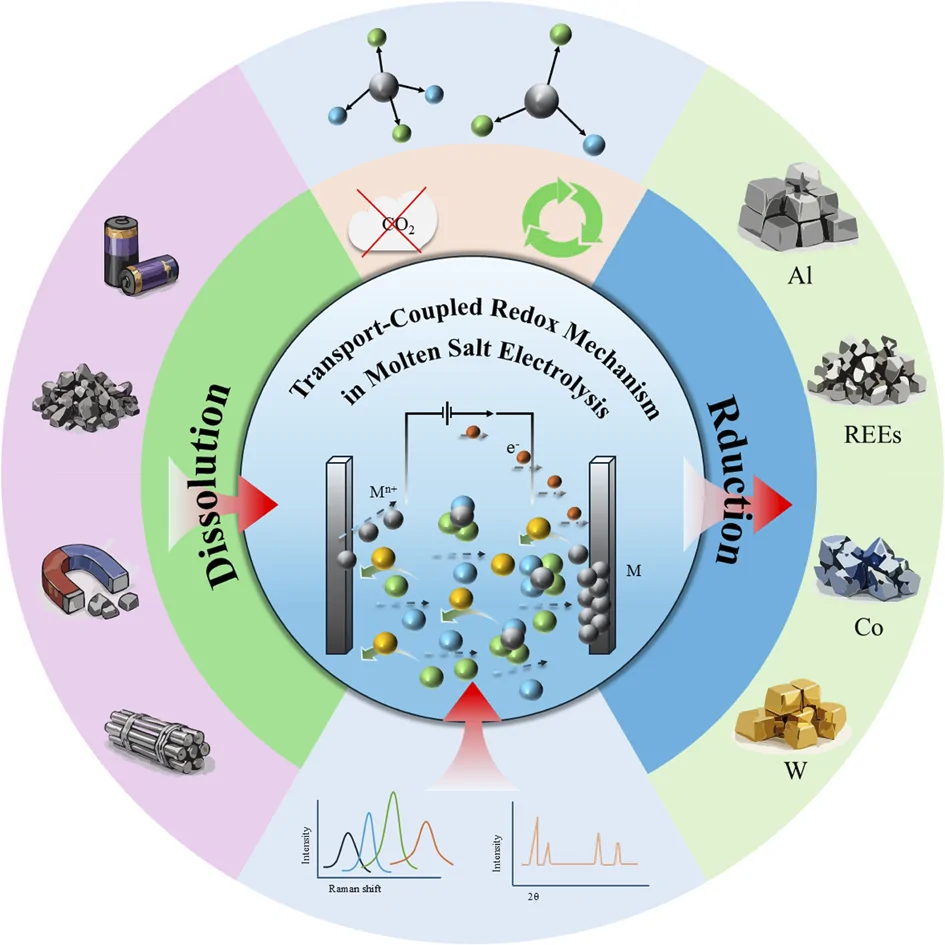

## 1 Introduction

In the context of secondary-resource recycling, critical and high-value metals can be broadly defined as metals that play irreplaceable roles in strategic industries, including advanced materials, new energy technologies, next-generation information technology, artificial intelligence, biotechnology, high-end equipment manufacturing, and national defense. These metals constitute the vital material foundation underpinning energy transition, technological innovation, and industrial upgrading.^1,2^

Recovering critical metals from secondary resources is motivated not only because of their resource value, but also because improper waste management poses serious risks to the environment and human health. Without effective recycling and reuse, such wastes may pose substantial long-term threats to ecosystems and public health. Improper disposal of spent lithium-ion batteries can lead to the release of toxic metals, including cobalt and nickel, into soils and aquatic environments. Spent nuclear fuel and zirconium alloy claddings contain long-lived radionuclides that require secure confinement, while recycling can substantially reduce waste volume and radiological hazards. In addition, metallurgical solid wastes, such as slag and red mud, may contain harmful elements including arsenic and fluorides, posing risks of leaching and airborne contamination. Therefore, the development and implementation of efficient recycling technologies, particularly molten salt electrolysis, are essential for achieving the sustainable recovery of critical metals while minimizing environmental impact.^3^

In this review, the term “critical and high-value metals” is used as a broader working definition rather than being restricted to a single jurisdictional list of critical minerals.^4,5^ The selection of metals considered here is based on four complementary criteria: (i) their recognized criticality or strategic importance in major economies;^4,5^ (ii) their importance to advanced manufacturing, energy transition, and other strategic applications;^5,6^ (iii) the availability and significance of corresponding secondary resources;^7^ and (iv) their relevance to the mechanistic characteristics of molten salt electrolysis.^8^ Accordingly, the review considers representative systems involving Li, Co, rare-earth elements, W, and Zr, while Al is included as a high-value and strategically important metal because of its large secondary-resource streams and the distinctive molten-salt coordination and electrochemical mechanisms involved in its recovery. This selection is therefore intended to provide mechanistically representative cases rather than to constitute an exhaustive list of all critical metals.

In the field of secondary metal resource recovery, traditional technologies, including pyrometallurgy, hydrometallurgy, and mechanical processing, have been widely implemented at industrial scale. Pyrometallurgy is particularly suitable for the large-scale treatment of compositionally complex secondary resources, whereas hydrometallurgy offers greater selectivity for the extraction and separation of low-abundance and high-value metals. Their complementary characteristics have also led to integrated routes combining pyrometallurgical pretreatment with subsequent hydrometallurgical refining. However, these established approaches face limitations in energy consumption, environmental impact, selectivity, and process complexity. Pyrometallurgical treatment generally requires high-temperature operation and intensive energy input, whereas hydrometallurgical routes often involve substantial reagent consumption and liquid-waste generation and may show limited efficiency for refractory phases. These limitations become particularly relevant for increasingly complex multimetallic secondary resources, motivating the development of alternative recovery strategies.^9,10^

Electrochemical metal extraction provides an alternative by using electrical energy directly to drive redox reactions. Depending on the reactivity of the target metal and the stability of the electrolyte, aqueous and molten-salt systems can be selected for different metallurgical applications. Aqueous electrolytes are generally suitable for the extraction and purification of less reactive metals, whereas molten salts provide an extended electrochemical window for the production and separation of highly reactive metals that are difficult to recover from aqueous media.^11^ In molten salt electrolysis (MSE), electrons serve as the reductant and the molten salt provides the ionic medium for metal-containing species, allowing their dissolution, transport, and electrochemical reduction within the same system.

Although extensive studies have explored molten salt systems for metal extraction, the interplay among molten-salt chemistry, mass transport, and interfacial electrochemical processes in secondary resource recovery remains insufficiently understood. This review therefore summarizes recent advances in MSE-based recovery of valuable metals from representative secondary resources, emphasizing electrochemical mechanisms and ionic-species evolution. MSE is considered as a transport-coupled electrochemical process involving feedstock dissolution, species coordination and migration, interfacial electron transfer, and metal reduction and deposition. As illustrated in Fig. 1, these interconnected processes constitute a unified mechanistic framework, whose controlling steps vary with feedstock composition, molten-salt chemistry, and electrode environment. The following sections apply this framework across different secondary-resource systems to identify their key chemical, transport, and interfacial processes.

**Fig. 1:**
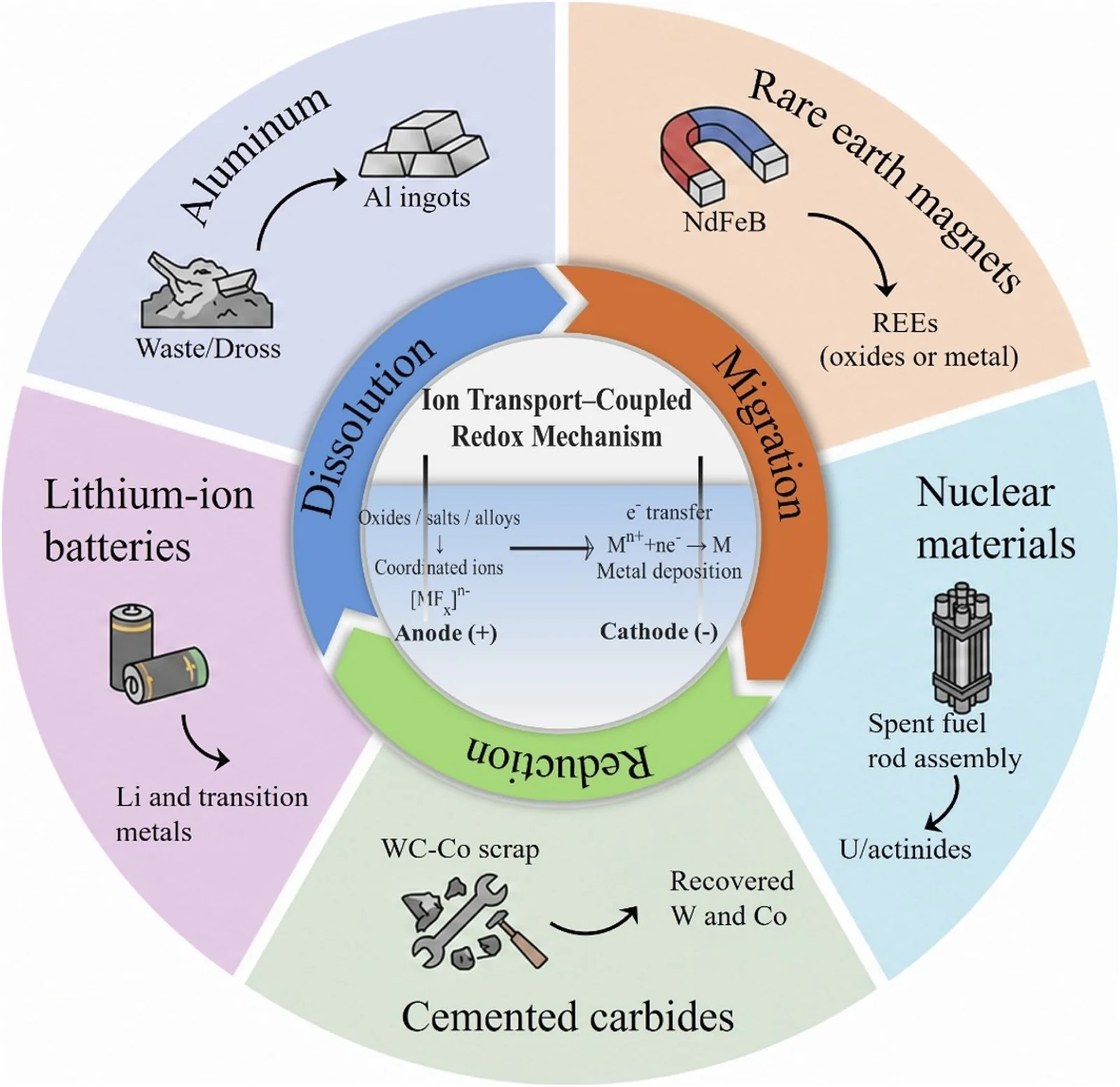
Mechanistic framework of molten salt electrolysis, linking coordination chemistry, dissolution, speciation, ionic transport, and electrochemical reduction to diverse secondary resource recovery pathways.

## 2 Mechanistic perspective of molten salt electrolysis for secondary critical metal recovery

Building on the unified framework introduced in Fig. 1, the mechanistic behavior of MSE can be further resolved into three closely coupled stages: feedstock activation and ionic-species evolution, ion transport and interfacial electron transfer, and subsequent reduction and deposition. The relative contribution of these processes depends strongly on the precursor phase, molten-salt chemistry, and electrode environment. In oxide-based resources, for example, electrochemical recovery may additionally involve progressive deoxygenation, solid-state diffusion, and oxygen-ion transport toward the anode. Understanding these coupled processes requires complementary evidence from spectroscopic characterization, phase and structural analysis, electrochemical measurements, and theoretical calculations, which together allow ionic evolution, reaction pathways, and rate-controlling steps to be distinguished.^12,13^

Although most secondary-resource recovery processes in molten salts can be described within a dissolution–migration–reduction framework, establishing the underlying mechanisms requires evidence linking ionic evolution, structural transformation, and interfacial electron transfer. Accordingly, complementary structural, spectroscopic, electrochemical, and computational approaches have been increasingly employed to distinguish reaction pathways and identify controlling steps.^14–19^

Structural characterization, including XRD, XPS, and electron microscopy, reveals phase evolution, changes in oxidation states, and morphological or interfacial reconstruction during electrolysis.^14,15,20^ Electrochemical techniques such as CV, chronoamperometry, and EIS provide complementary information on redox behavior, mass transport, nucleation, and charge-transfer kinetics.^16,17,19^ Equivalent-circuit analysis of EIS spectra can further deconvolute electrolyte and interfacial contributions, with charge-transfer resistance and Warburg impedance providing quantitative indicators of interfacial kinetics and mass transport, respectively.^21^ In molten-salt electrodeposition, more detailed impedance models can additionally distinguish charge-transfer, surface-diffusion, and lattice-formation resistances, thereby providing quantitative insight into the kinetic limitations associated with interfacial deposition and subsequent incorporation into the growing metal phase.^22^ Recent electrodeposition studies further demonstrate that electrochemical responses can be correlated with reduction processes, electrolysis efficiency, and deposition behavior to elucidate interfacial reduction mechanisms.^19,23^ Their correlation with structural changes is particularly useful for identifying transient intermediates and distinguishing electrochemical reactions from concurrent thermal or chemical transformations.

Because molten salts operate at elevated temperatures, *in situ* and *operando* techniques are particularly important for resolving transient ionic structures. As illustrated in Fig. 2, Raman spectroscopy, XANES, EXAFS, and *in situ* UV-vis spectroscopy can probe coordination environments, oxidation states, and their evolution under operating conditions, while AES-based approaches can provide complementary information on changes in ionic concentrations.^18,20^ These measurements help establish direct links between ionic speciation and electrochemical behavior that are difficult to obtain from *ex situ* characterization alone.

**Fig. 2:**
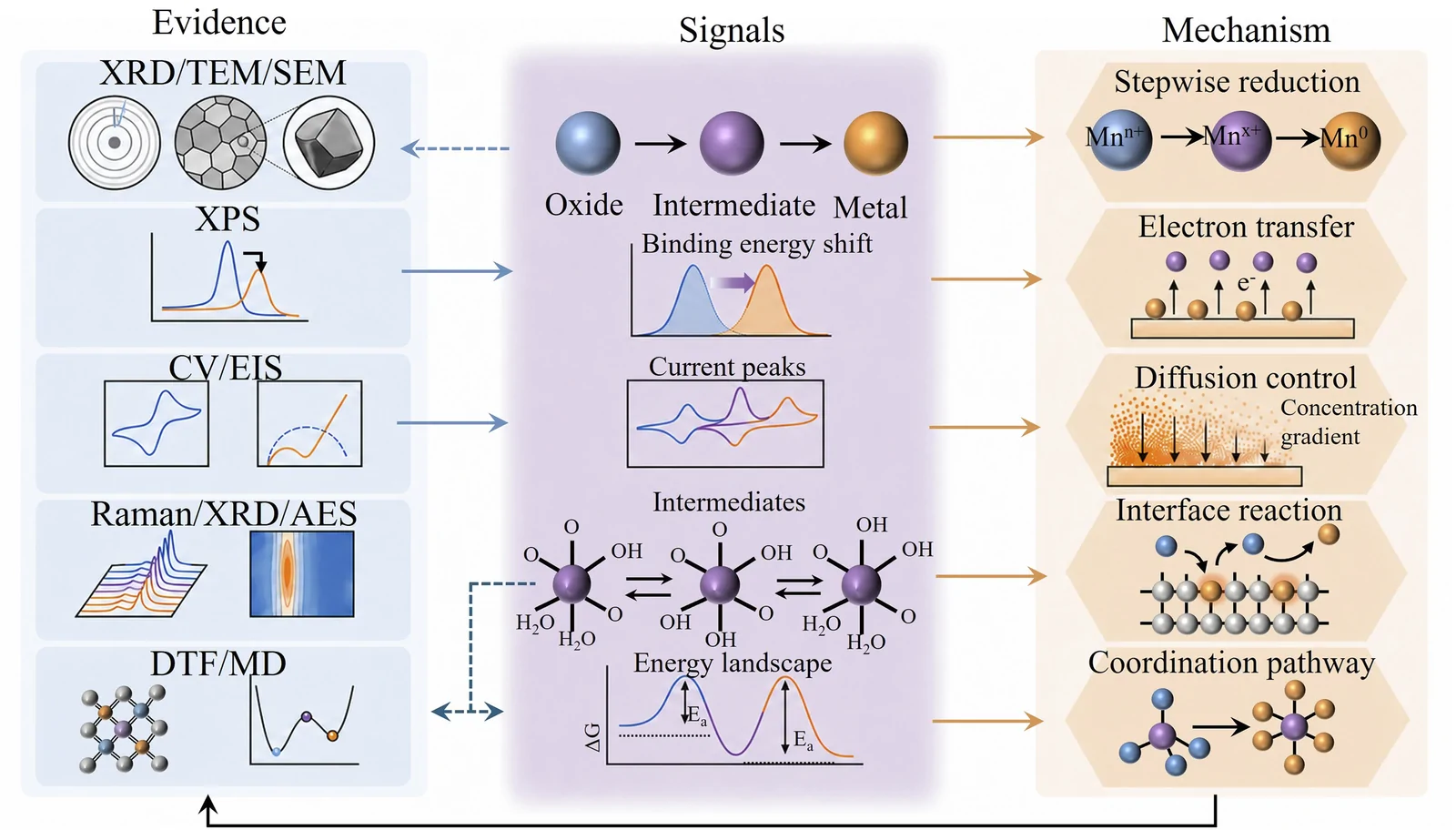
Correlated validation of molten salt mechanisms through multi-scale probes of coordination structure, oxidation state, and dynamic evolution.

Computational methods further complement experimental observations by providing atomic- and electronic-level insight into ion coordination, electrode–electrolyte interactions, and reaction energetics. DFT, molecular dynamics, and first-principles molecular dynamics can elucidate reaction mechanisms and coordination evolution, while machine-learning-based potentials extend simulations to larger and more complex molten-salt systems.^24–26^ Collectively, these approaches transform the dissolution–migration–reduction framework from a conceptual description into an evidence-based mechanistic model.

Beyond the conventional description of ionic speciation and electrochemical kinetics, the local physicochemical environment of the electrolyte–electrode interface can also influence ion transport and charge collection. In particular, the local dielectric environment may alter electrostatic interactions between ionic species and the energy required for ionic rearrangement during migration, thereby influencing ion-transport barriers and interfacial charge transfer. These effects are particularly relevant to molten-salt processing of LIB-derived materials, where Li-ion transport, cathode structural evolution, and electrode/electrolyte interactions are strongly coupled.

## 3 Mechanisms of aluminum from secondary resources in molten salt systems

Secondary aluminum resources, including aluminum dross, scrap aluminum alloys, and coal fly ash, represent important reservoirs of aluminum that can be reutilized using molten salt technology. With the rapid growth in global aluminum demand and the increasing need for low-carbon metallurgical processes, the recycling of secondary aluminum resources is attracting growing attention.^27,28^ Compared to primary aluminum production, which relies heavily on energy-intensive Hall–Héroult electrolysis and generates significant greenhouse gas emissions,^29,30^ molten salt recycling technologies offer advantages such as lower operating temperatures, flexible feedstock tolerance, and selective electrochemical reactions.

Based on the physicochemical properties of the raw materials, the mechanisms for recovering aluminum from secondary resources can generally be categorized into oxide reduction electrolysis, electrorefining, and mineral phase reconstruction coupled with molten salt reduction.^31,32^

### 3.1 Aluminum dross

Aluminum dross is a byproduct generated during smelting and recycling processes. Its composition includes metallic aluminum dispersed within refractory phases such as Al_2_O_3_, AlN, and MgAl_2_O_4_, which impose substantial kinetic limitations for metal recovery.^31,33–35^ Li *et al.*^36^ showed that reactive phases in secondary aluminum dross undergo transformation during treatment, facilitating aluminum release. The evolution of aluminum recycling practices, including advanced scrap sorting and next-generation vehicle-driven material flows, has increased the availability of such secondary resources and highlighted the need for efficient recovery strategies.^37^ Within the unified mechanistic framework established above, aluminum dross is particularly influenced by the heterogeneous and refractory nature of its Al-bearing phases. As illustrated in Fig. 3(a), refractory oxide phases can restrict the penetration of the molten electrolyte into the particle interior, leaving an unreacted core and limiting the generation and transport of electrochemically accessible Al-containing species. Metallic aluminum droplets can segregate from the molten salt based on density, whereas aluminum trapped in refractory oxides requires dissolution to generate electrochemically accessible species.^38^

**Fig. 3:**
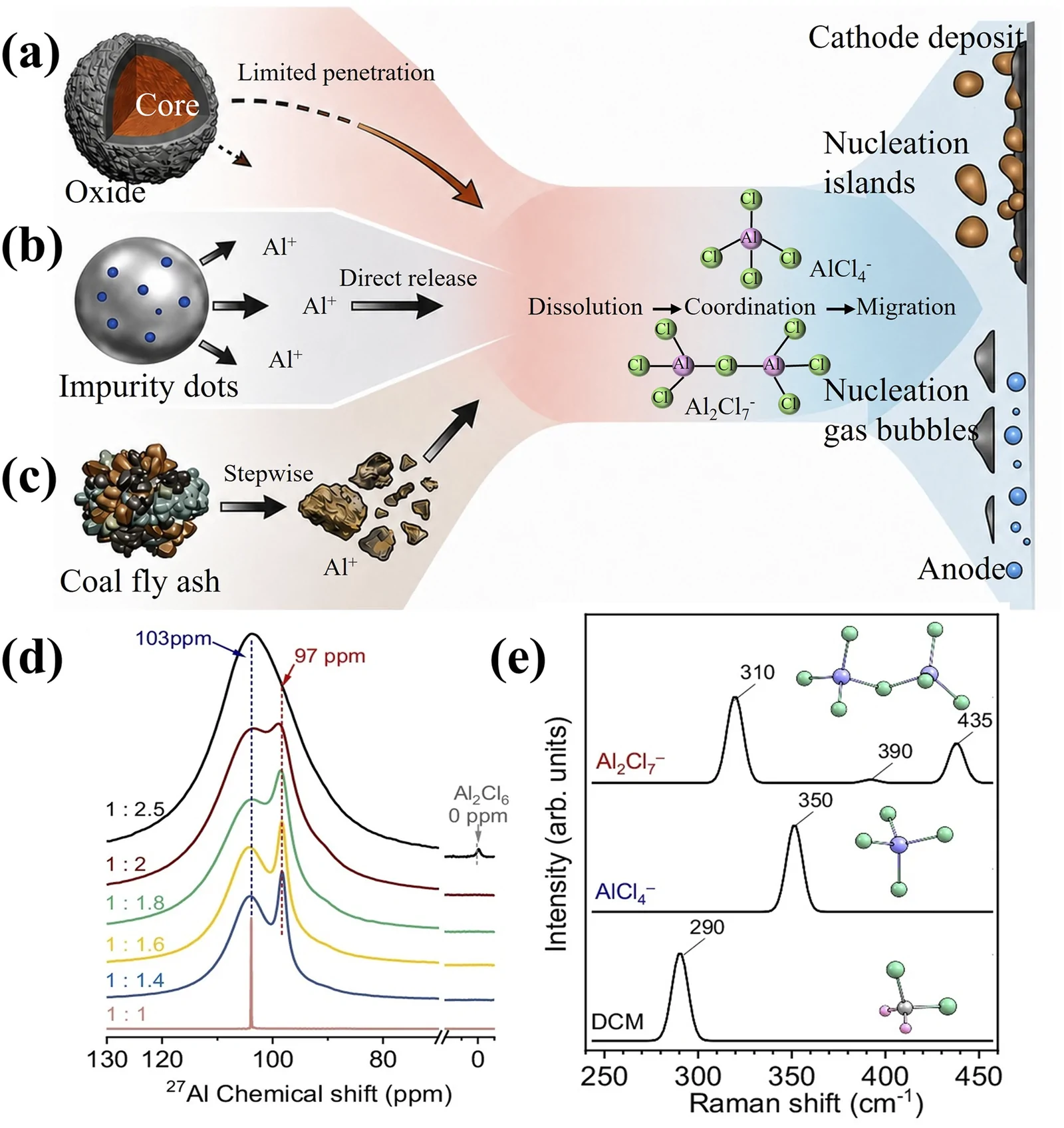
Mechanistic pathways and molecular-level evidence for aluminum recovery from secondary resources in molten salt systems. (a) Limited penetration of the molten electrolyte into refractory oxide particles, resulting in an unreacted core; (b) direct release of Al-containing species from impurity-containing solid matrices followed by dissolution, coordination, and migration of chloroaluminate species toward the cathode; (c) stepwise fragmentation and Al-containing phase release from coal fly ash; (d) Al MAS NMR spectra of [C_4_Py]Cl-*x*AlCl_3_ (*x* = 1–2.5). Reprinted with permission from ref. 73 (e) DFT-simulated Raman spectra of AlCl_4_^−^, Al_2_Cl_7_^−^, and DCM. Reprinted with permission from ref. 73. This figure has been adapted from the original source and reproduced in accordance with the applicable open-access license.

In chloride–fluoride molten systems, dissolution of Al_2_O_3_ establishes equilibria involving chloroaluminate species such as AlCl_4_^−^ and Al_2_Cl_7_^−^, thereby controlling the concentration and availability of electroactive aluminum species at the cathode.^39–41^ As illustrated in Fig. 3(b), Al-containing species released from the solid matrix undergo dissolution and coordination reconstruction to form chloroaluminate complexes, followed by migration toward the cathode. This sequence links the release of Al from heterogeneous solid phases with the formation and transport of electroactive species before cathodic deposition. The cathodic reaction consequently proceeds through coordinated chloroaluminate complexes rather than free Al^3+^ ions.^41,42^ The distribution of these species depends on molten-salt composition and influences the subsequent electron-transfer process and reduction kinetics. Electrolyte additives can further modify charge-transfer behavior and reaction pathways by altering the coordination environment and ionic structure of the melt.^39–41^ When oxide dissolution is insufficient, undissolved particles accumulate and restrict the supply of electroactive species, resulting in transport-limited reduction. Beyond the intrinsic dissolution kinetics of refractory phases, the rheological properties of molten electrolytes can influence oxide dissolution and mass transport. Molecular dynamics simulations of NaF–AlF_3_ melts showed that the viscosity increased from 1.61 to 2.57 mPa s as the cryolite ratio (CR) increased from 2.0 to 3.0, corresponding to an increase of approximately 59.6%.^43^ In a separate experimental study, the addition of NaF, KF, and CaF_2_ (up to 5 wt%) to equimolar NaCl–KCl fluxes decreased the melt viscosity at 760 and 810 °C and increased the extent of alumina-rod reduction at 750 °C.^44^ These findings show that electrolyte composition can affect both melt viscosity and alumina dissolution behavior. Increased viscosity may suppress convective mixing and slow electrolyte renewal at solid–liquid interfaces, restricting the transport of dissolved aluminum species and the accessibility of oxide-bearing surfaces. In heterogeneous aluminum dross, these effects may hinder electrolyte penetration and oxide dissolution, thereby increasing the contribution of mass transport to the overall recovery process. Consequently, oxide dissolution, aluminum speciation, and cathodic reduction are coupled through both the availability of electroactive species and the transport properties of the melt.

The anodic pathway depends on the electrode material: an inert anode releases oxygen eqn (1), whereas a carbon anode produces CO/CO_2_*e.g.*, eqn (1) and (2). Ueda *et al.*^45^ highlighted that overall kinetics are not solely controlled by interfacial charge transfer but are strongly influenced by oxide dissolution rates, ion migration, and structural accessibility. The anodic gas-evolution pathway can therefore be considered an auxiliary interfacial factor within this mechanism. Gas bubbles undergo nucleation, growth, coalescence, and detachment, forming a transient gas–electrolyte boundary layer near the anode. Persistent bubble coverage can partially block the electrode surface, reduce the effective electrochemically active area, redistribute the local current density, and increase ohmic losses. In cryolite-alumina melts, the average bubble coverage at 0.7 A cm^−2^ was reported to be 49.93% on carbon anodes and 61.19% on graphite anodes, while the maximum coverage reached 73.54% and 87.83%, respectively, demonstrating the influence of anode properties on bubble coverage.^46^ Meanwhile, bubble-driven liquid motion can alter local convection and mass transfer, thereby affecting the transport of oxygen-containing species through the electrolyte.^46,47^ Anode structural design may help mitigate these effects. For example, inclined or slotted anodes can facilitate bubble detachment and directional gas evacuation, whereas the effectiveness of porous or mesh-type structures requires evaluation under the specific conditions of molten-salt electrolysis.^47,48^ In Ca–CaCl_2_ melts, in addition to direct electroreduction pathways, chemically assisted mechanisms may also play a role. For example, in Ca-containing melts, Yan and colleagues^49^ demonstrated that electrochemically generated calcium acts as a transient reductant, enabling the reduction of Al_2_O_3_*via* a chemically-assisted electrochemical pathway:

1 2O^2−^ → O_2_ + 4e^−^

2 2O^2−^ + C → CO_2_ + 4e^−^

3 C + O^2−^ → CO + 2e^−^

4 Al^3+^(coord) + 3e^−^ → Al(l)

5 Al_2_O_3_ + 3Ca → 2Al + 3CaO

This mechanism underscores that aluminum reduction is not purely chemical but a dynamically sustained process controlled by electron transfer at the cathode. Due to the high thermodynamic stability of AlN, it preferentially reacts at three-phase boundaries involving solid particles, molten electrolyte, and the electrode, thereby limiting the overall conversion rate.^31,49^ Pre-treatment strategies, such as hydrolysis to Al(OH)_3_ followed by calcination to Al_2_O_3_, have been shown to transform inert phases into electrochemically accessible species, thereby enhancing reduction kinetics.^49^

At the microstructural level, the reduction process exhibits a diffusion-coupled shrinking-core behavior. Yan *et al.*^49^ observed that the reaction front advances inward from particle surfaces, generating porous shells that modulate ion accessibility. Wang *et al.*^31^ further emphasized that this structural evolution, in conjunction with dissolution and ion migration, collectively determines the reaction's trajectory and efficiency. The progressive formation of porous reaction layers therefore couples phase conversion with mass transport, making particle-scale structural evolution an important kinetic factor.

Quantitative electrochemical analysis further helps distinguish transport and interfacial contributions to the reduction step. In molten AlCl_3_–NaCl–KCl, Jafarian *et al.*^22^ used an equivalent-circuit model to separate solution/electrode resistance (*R*_1_), charge-transfer resistance (*R*_2_), surface-diffusion resistance, and lattice-formation resistance (*R*_3_ and *R*_4_). At cathodic potentials from −0.82 to −0.86 V *vs.* Al, *R*_1_ remained approximately constant at 11 Ω, whereas *R*_2_ decreased from 18.6 to 4 Ω and *R*_3_ + *R*_4_ decreased from 68 to 12 Ω, indicating that increased cathodic driving force progressively facilitates interfacial charge transfer and subsequent surface/lattice incorporation of Al. Similar impedance-based separation of mass-transfer and kinetic contributions has been demonstrated during uranium recovery in molten LiCl–KCl.^21^ These results show that EIS can quantitatively distinguish transport- and reduction-related contributions within the migration–reduction stage, while the preceding structural activation and dissolution of refractory Al-bearing phases remain essential for generating electrochemically accessible Al species.

FTIR analysis revealed –OH stretching bands at 3616, 3552, 3427, and 3370 cm^−1^ in the hydrolysis residue, while a characteristic Al–O–Al vibration at 570 cm^−1^ emerged after calcination. Combined with XRD analysis, these results demonstrated the progressive conversion of AlN to γ-Al_2_O_3_ through hydrolysis and calcination.^50^ Further experiments showed that a dense Al–O layer of approximately 25–45 nm formed on the AlN surface. After treatment at 900 °C for 150 min, the oxidation degree was only 19% without additives, but increased to 78%, 86%, and 99% with the addition of NaF, AlF_3_, and Na_3_AlF_6_, respectively, indicating that surface passivation is a key barrier to the conversion of refractory AlN.^51^ DFT calculations further yielded O_2_ adsorption energies of −72.78 and 6.03 kcal mol^−1^ on different AlN surfaces, with corresponding dissociation barriers of 7.57 and 10.08 kcal mol^−1^. DPMD simulations showed that the dense [Al–O] layer suppresses O_2_ diffusion into the AlN interior, providing an atomistic explanation for the kinetically inhibited conversion.^52^ Ball-milling pretreatment reduced the activation energy for AlN oxidation by approximately 67 kJ mol^−1^, further demonstrating that structural activation can substantially lower the kinetic barrier.^53^ Electrochemical studies showed that, in the AlF_3_–NaF–BaCl_2_ molten salt, 83% of metallic Al could be recovered by flotation, while an additional 4% was recovered by electrolysis of the separated oxide fraction; continuous electrodeposition was constrained by the balance between oxide dissolution and electrodeposition rates.^51^ In GaCl_2_-based molten salt, Al_2_O_3_ underwent appreciable reduction, whereas direct reduction of AlN was limited by the three-phase interface and its high thermodynamic stability.^49^

Taken together, these quantitative observations establish a continuous kinetic chain: disruption of the refractory Al–O passivation layer facilitates structural activation and dissolution; the resulting increase in electrochemically accessible Al-containing species supports ionic migration; and the subsequent decrease in charge-transfer and deposition-related resistances under cathodic polarization facilitates interfacial reduction. Collectively, these quantitative results establish a mechanistic sequence in which structural activation and disruption of the passivating Al–O layer facilitate the conversion and dissolution of refractory AlN-derived phases, thereby enabling subsequent electrochemical reduction, with the overall process governed by interfacial reaction and mass-transfer kinetics.

### 3.2 Aluminum scrap

Aluminum alloy scrap is an important source of secondary aluminum and typically contains alloying elements such as Si, Fe, Mg, and Mn.^52,55,56^ The presence of these impurities complicates conventional remelting processes, making molten salt electrolysis a highly effective approach for selective aluminum recovery. In this process, aluminum preferentially dissolves from the alloy anode due to its lower anodic dissolution potential, governed by the fundamental electrochemical reactions:

6 Anode: Al → Al^3+^ + 3e^−^

7 Cathode: Al^3+^ + 3e^−^ → Al

Within the unified mechanistic framework, the distinctive feature of aluminum alloy scrap is the coupling between selective anodic dissolution and coordination-controlled cathodic deposition. Jafarian *et al.*^57^ demonstrated that aluminum electrodeposition from AlCl_3_–NaCl–KC molten salts follows a nucleation-and-growth mechanism involving chloroaluminate complexes. Coordinated AlCl_4_^−^ and Al_2_Cl_7_^−^ species regulate electron transfer and subsequent surface deposition. Raman spectroscopy confirms the coexistence and dynamic equilibrium of these species in ionic melts.^39,58^ Guo *et al.*^54,55^ further showed that aluminum-containing species migrate toward the cathode, where their reduction and the resulting deposit morphology depend strongly on local ion flux, nucleation density, and electrolyte composition.

For Mg-containing alloys, the reduction pathway becomes more complex. Sun *et al.*^59^ observed underpotential deposition of Mg during the initial electrolysis stage, followed by Mg–Al co-deposition, before Al deposition became dominant as Mg reduction was suppressed. This behavior demonstrates that alloy composition can modify the cathodic stage of the unified framework by introducing competing deposition pathways and transient co-deposition. Selective anodic dissolution of Al while impurity elements are retained in the alloy anode therefore contributes to the high-purity recovery of aluminum.

The stability of electrode and containment materials also affects the long-term integrity of the metal–electrolyte interface. Under molten-fluoride aluminum electrolysis conditions, a Cu–Ni-based alloy exhibited a reported corrosion rate of 12.7 mm per year at 960 °C and 0.5 A cm^−2^. In a separate study conducted at 700 °C in a KF–AlF_3_ electrolyte, a Cu–Ni–Fe alloy without Fe developed an unstable voltage of approximately 6 V, and the resulting aluminum contained 0.92 wt% Cu and 0.12 wt% Ni. Increasing the Fe content to 15 wt% reduced the corresponding Cu and Ni contamination to 0.39 wt% and 0.10 wt%, respectively, while stabilizing the electrolysis voltage at approximately 3.7–4.4 V.^60^ These results demonstrate that alloy composition and protective oxide-scale stability can influence corrosion, interfacial integrity, electrolysis stability, and aluminum purity. However, the reported values are specific to the respective alloy compositions and operating conditions and should not be interpreted as universal corrosion rates or contamination levels.

The coordination environment of the molten salt further determines the cathodic reaction pathway. The ^27^Al MAS NMR spectra shown in Fig. 3(d) reveal systematic changes in the local Al chemical environment with increasing AlCl_3_ content, as reflected by the evolution of the characteristic resonances from approximately 103 to 97 ppm. These changes indicate that the coordination and speciation of Al-containing species are strongly dependent on the composition of the chloride melt. Böttcher *et al.*^65^ identified characteristic vibrational bands corresponding toAlCl_4_^−^ and Al_2_Cl_7_^−^ in AlCl_3_-based ionic liquids, confirming the coexistence of monomeric and dimeric chloroaluminate species and their dynamic equilibrium. These chloroaluminate species, AlCl_4_^−^ and Al_2_Cl_7_^−^, act as the primary electroactive centers and enable aluminum reduction to proceed *via* a coordination-controlled mechanism involving species-dependent electron transfer. Molecular-level models demonstrate that coordination environment modulates the energy barrier for reduction and surface nucleation steps in molten salt systems.^40,58^ undergoing successive reduction at the cathode:

8 Al_2_Cl_7_^−^ + 3e^−^ → 2Al + 7Cl^−^

9 AlCl_4_^−^ + 3e^−^ → Al + 4Cl^−^

The proposed mechanism of selective anodic dissolution–coordination reconstruction–cathodic deposition is supported by converging spectroscopic, electrochemical, and computational evidence. Spectroscopic studies have identified chloroaluminate species such as AlCl_4_^−^ and Al_2_Cl_7_^−^, together with their characteristic vibrational features associated with Al–Cl coordination, supporting the coordination-controlled speciation proposed in the mechanistic framework.^61,62^ Electrochemical investigations further demonstrated that Al preferentially dissolves from Al–Si alloys at the anode, accompanied by Si enrichment at the anode surface, while the deposited Al reached a purity above 99.4 wt%, with some alloys yielding up to 99.9 wt% Al.^63^ In molten-salt electrolysis of waste Al alloys, Al dissolution initiated at approximately −1.6 V, whereas Fe underwent electrochemical dissolution at around −0.9 V, ultimately enabling Al recovery with 99.8% purity.^52^ These potential-dependent behaviors provide direct evidence for selective anodic dissolution among alloy constituents. Complementary computational studies reproduced the optimized structures and corresponding IR vibrational features of AlCl_4_^−^ and Al_2_Cl_7_^−^, in agreement with experimental observations. As shown in Fig. 3(e), the DFT-simulated Raman spectra exhibit characteristic bands at approximately 350 cm^−1^ for AlCl_4_^−^ and 310, 390, and 435 cm^−1^ for Al_2_Cl_7_^−^, providing molecular-level theoretical support for the assignment of these chloroaluminate species. The agreement between calculated vibrational features and spectroscopic observations further supports the proposed coordination structures and their role in governing cathodic Al reduction.^62,64^ Collectively, these results establish a mechanistic sequence in which alloy constituents undergo potential-selective anodic dissolution, followed by coordination/speciation reconstruction in the molten salt and subsequent cathodic Al deposition, rather than undergoing indiscriminate dissolution and reduction.

### 3.3 Coal fly ash

Coal fly ash is a major byproduct of coal combustion and contains a large amount of aluminum, primarily in the form of Al_2_O_3_ and aluminosilicate minerals such as mullite (3Al_2_O_3_·2SiO_2_), along with minor Fe and Ti oxides.^65,66^ The chemical inertness of these phases limits direct electrochemical reduction, requiring the transformation of aluminum-containing minerals into electrochemically accessible species before electrodeposition. Within the unified mechanistic framework, the distinctive feature of coal fly ash is therefore the strong coupling between mineral activation and coordination reconstruction. As illustrated in Fig. 3(c), the heterogeneous coal-fly-ash matrix undergoes stepwise fragmentation and release of Al-containing phases, which facilitates their subsequent dissolution, coordination reconstruction, and migration in the molten electrolyte. This stepwise release distinguishes coal fly ash from relatively more accessible aluminum-containing feedstocks and highlights mineral-phase activation as a prerequisite for efficient electrochemical recovery.

Liu *et al.*^67^ demonstrated that in NaF–AlF_3_ melts, thermally pretreated coal fly ash could be partially converted into Al–Si intermediate alloys, with aluminum forming soluble hexafluoroaluminate complexes. Complementary studies in chloride-based melts show that aluminum forms coordinated species, AlCl_4_^−^ and Al_2_Cl_7_^−^, which determine the electroactive species and reduction kinetics, reinforcing the general role of coordination in cathodic processes.^65^

10 Al_2_O_3_ + 6F^−^ → 2AlF_6_^3−^

These complexes subsequently undergo cathodic reduction:

11 AlF_6_^3−^ + 3e^−^ → Al + 6F^−^

Although these reactions highlight the importance of fluoroaluminate coordination, eqn (10) represents a simplified speciation process rather than an elementary dissolution pathway, as multiple oxyfluoride and fluoroaluminate species may coexist. More importantly, soluble Al–F complexes do not necessarily ensure efficient recovery, because their stability, transport, interfacial reduction, and interactions with Si and impurities collectively determine current efficiency and selectivity.

Fluoride systems thus involve a fundamental trade-off: strong Al–F complexation facilitates refractory-phase activation, but high-temperature operation also causes severe corrosion, environmental concerns, and demanding materials requirements. For coal fly ash, the high SiO_2_ content and complex mineralogy may further increase electrolyte complexity without proportionally improving electrochemically recoverable Al. Therefore, high Al conversion alone does not demonstrate process viability or scalability.

Chloride-based systems have been explored to mitigate these limitations. Li *et al.*^68^ showed that CaCl_2_-assisted pre-calcination promotes Al_2_O_3_ dissolution and enables deposition of Al–Si alloys containing minor Fe and Ti. Johnston *et al.*^69^ proposed an integrated acid-leaching–chlorination–electrolysis route, in which Al_2_O_3_ is first converted to AlCl_3_ before molten chloride electrolysis:

12 Al_2_O_3_ + 6HCl → 2AlCl_3_ + 3H_2_O

13 2AlCl_3_ → 2Al + 3Cl_2_

However, chloride media should not simply be regarded as a more practical replacement for fluoride systems. Although their lower operating temperatures can alleviate some corrosion concerns, AlCl_3_ volatility may cause electrolyte loss, alter Al speciation, and complicate off-gas management. Incomplete dissolution and competing reactions involving Fe, Si, and other impurities can further limit current efficiency. Thus, chloride systems shift rather than eliminate the key bottlenecks, with volatility, impurity tolerance, current efficiency, and electrolyte stability remaining major scale-up challenges.

The proposed “mineral-phase activation–coordination reconstruction–electrochemical reduction” mechanism is supported by complementary spectroscopic, electrochemical, and computational evidence. In an Al_2_O_3_–Na_3_AlF_6_ melt with a chemical environment analogous to that of alumina in coal fly ash, *in situ* high-temperature Raman spectroscopy identified characteristic bands of AlF_4_^−^ AlF_5_^2−^, and AlF_6_^3−^ at approximately 622, 555, and 515 cm^−1^, respectively. With increasing Al_2_O_3_ concentration, Al_2_OF_6_^2−^ increased progressively, while Al_2_OF_6_^2−^ emerged above 3 wt% Al_2_O_3_, indicating pronounced Al–O–F/Al–F coordination reconstruction during oxide dissolution; first-principles calculations further reproduced the structures and vibrational characteristics of these species.^70^ In direct coal-fly-ash electrolysis, Tao *et al.*^71^ showed from cyclic voltammetry and electrodeposition experiments that Si(iv) undergoes multistep electrochemical reduction, with combined Al and Si recovery efficiencies of 87.33% and 84.02%, respectively. Further studies showed that Al_2_O_3_ promotes NaAlSi_3_O_8_ formation, increasing the dissolution rate by 2.4-fold and reducing Si loss from 25.84% to 0.70%; electrochemical analysis further indicated an electrochemical–aluminothermic reduction pathway, yielding Al and Si recoveries of 94.55% and 98.31%, respectively.^72^ Collectively, these results demonstrate that coal-fly-ash conversion in molten salts proceeds through mineral-phase activation and Al–O–F/Al–Si coordination reconstruction, followed by multistep interfacial reduction, rather than by direct reduction of refractory oxides.

Molten salt electrolysis provides a versatile route for aluminum recovery from diverse secondary resources, including aluminum dross, scrap alloys, and coal fly ash. Although these systems share the same fundamental electrochemical framework, their controlling mechanisms differ according to feedstock composition and structure. For aluminum dross, refractory-phase dissolution and microstructure-dependent mass transport strongly influence the generation and accessibility of electroactive Al species. For aluminum alloy scrap, selective anodic dissolution and chloroaluminate speciation govern subsequent cathodic deposition, while alloying elements can further affect the deposition behavior. For coal fly ash, mineral activation and coordination reconstruction are particularly important for converting refractory aluminosilicate phases into electrochemically accessible Al complexes.

The complex composition of secondary aluminum resources therefore remains a major challenge. Refractory phases such as AlN and mullite can limit aluminum dissolution, while impurity species may modify coordination environments, reduction behavior, and deposit quality. Future optimization should consequently consider feedstock activation, electrolyte speciation, mass transport, and nucleation behavior in an integrated manner. A mechanistic understanding of these coupled processes is essential for improving the selectivity, efficiency, and scalability of molten salt electrolysis for complex secondary aluminum resources.

## 4 Mechanisms of rare-earth recycling from NdFeB magnets in molten salts

This section elucidates the fundamental mechanisms underlying the recovery of rare-earth elements (REEs) from spent NdFeB magnets in molten salt systems, emphasizing the intrinsic coupling between thermodynamic activation, electrochemical reduction, and dynamic phase evolution. Rather than proceeding independently, these processes operate within a unified reaction network in which the formation of halides, electroreduction, and slag reconstruction are interdependent. Recent studies have further highlighted the importance of sustainable recovery strategies for spent NdFeB magnets, emphasizing the need to integrate resource efficiency and environmental considerations into rare-earth supply chains.^74–78^

At a more fundamental level, rare-earth species typically exist as halide-coordinated complexes rather than free ions, and their dynamic coordination states determine their solubility, mobility, and electrochemical activity.^76,79–81^ Consequently, there is an intrinsic relationship among melt structure, ion coordination, and reduction pathways, which forms the basis of REE recovery mechanisms.^79,81,82^

### 4.1 Oxide destabilization and coordination-mediated electroreduction pathways

Recovering rare earth elements from spent NdFeB magnets requires destabilizing refractory rare-earth oxides and converting them into electrochemically accessible species.^83–85^ Within the unified mechanistic framework, rare-earth recovery is characterized by the coexistence and competition between direct solid-state electroreduction and dissolution–electroreduction mediated by coordinated rare-earth species. The relative contribution of these pathways is governed by electrolyte composition, temperature, oxide stability, and interfacial conditions.

In fluoride-rich molten salts, relatively insoluble rare-earth oxides can undergo direct electrochemical deoxygenation at the oxide–electrolyte interface:

14 RE_2_O_3_ + 6e^−^ → 2RE + 3O^2−^

Electrochemical studies have reported cathodic reduction responses and progressive reduction behavior during rare-earth oxide electrolysis, indicating that oxygen removal is coupled with solid-state transport and interfacial charge transfer.^78,81^ Fluoride additives can additionally promote conversion of RE-O phases into RE-F phases. XRD and EPMA confirmed NdF_3_ formation during the fluorination of Nd_2_O_3_, demonstrating that halide-assisted phase conversion can provide an alternative route for destabilizing refractory oxides.^86^

With increasing chloride content, dissolution–electroreduction becomes more favorable because rare-earth species can be transferred from the solid phase into the molten salt. NdFeB-derived rare earths were converted into chloride species and transferred into MgCl_2_–KCl melts, achieving extraction efficiencies above 90% under optimized conditions.^79^ The dissolved rare-earth species subsequently undergo dynamic halide coordination and are reduced at the cathode:

15 RE^3+^ + 3e^−^ → RE

In NdCl_3_–LiCl–KCl melts, Nd electrolysis achieved a coulombic efficiency above 80% and a metal purity above 97 wt%.^77^ These results establish a direct link between rare-earth dissolution in halide melts and subsequent metal deposition.

The structural basis for this pathway transition is further supported by atomistic simulations. Fig. 4 illustrates the coordination restructuring of molten salt networks induced by dissolved multivalent metal species. FPMD calculations for LiF–NdF_3_ melts revealed concentration-dependent changes in Nd–F coordination and ionic-cluster structures, indicating that melt composition regulates the local coordination environment and ion-transport behavior of dissolved rare-earth species.^87^ Consistent with this compositional effect, the viscosity of (NaF–KF)_eut_–NdF_3_ melts increased with increasing NdF_3_ concentration. Rudenko *et al.* reported an approximately twofold increase in viscosity upon the addition of 15 mol% NdF_3_.^88^ This increase in viscosity may impede convective mixing and electrolyte renewal near the electrode surface, thereby affecting the mass transport of electroactive species during rare-earth recovery. The corresponding structural evolution is illustrated in Fig. 4(b) and (c), where Raman spectra of quenched Gd_2_O_3_–FLiNaK samples treated at different temperatures and XRD patterns of the quenched products collectively provide evidence of changes in the local coordination environment and phase composition of the molten-salt system. Thus, direct oxide electroreduction and dissolution-mediated reduction should not be regarded as mutually exclusive mechanisms; rather, changes in halide composition can shift the balance between interfacial reduction of refractory oxides and the dissolution, coordination, transport, and subsequent reduction of rare-earth species.

**Fig. 4:**
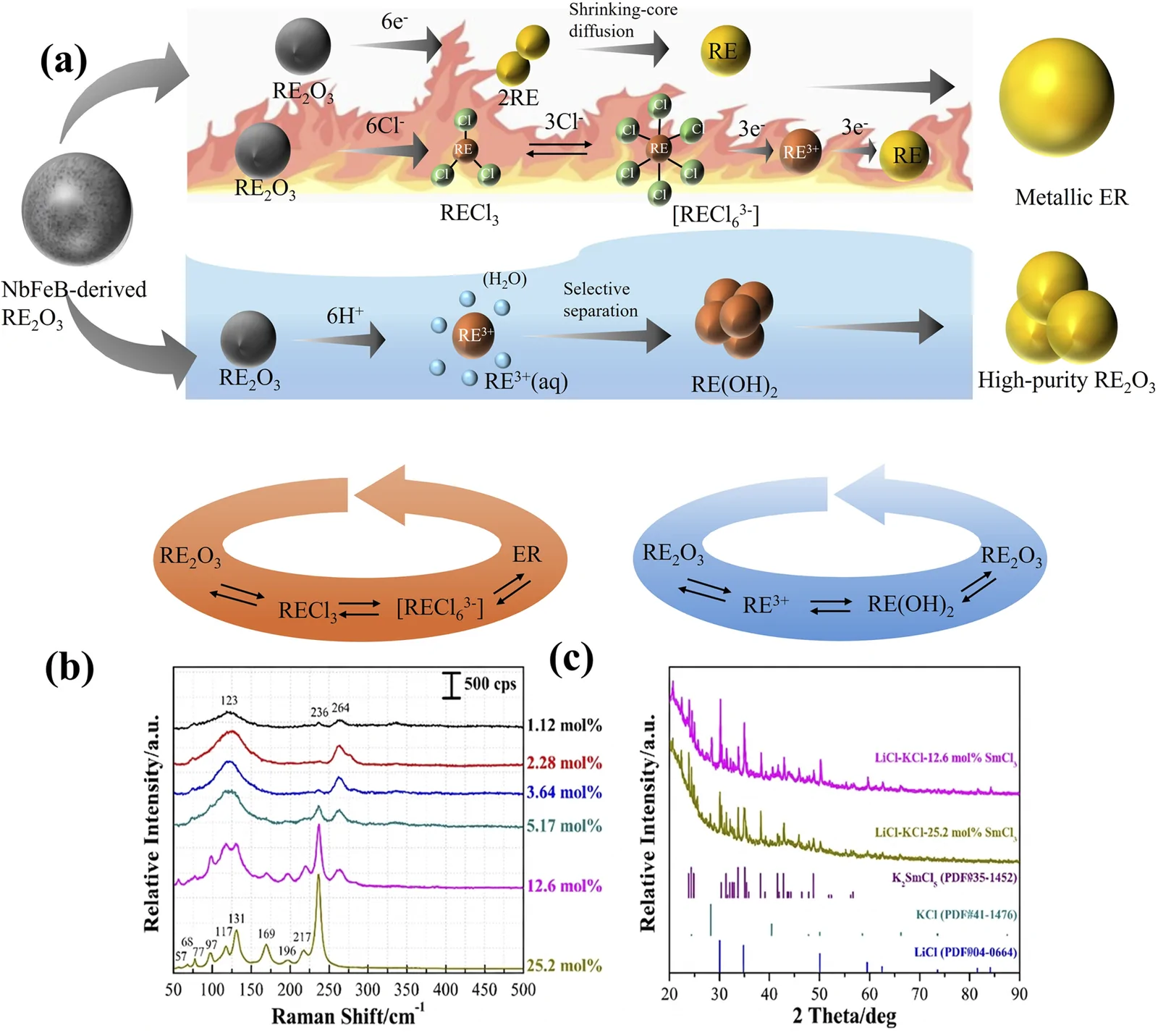
(a) Coordination restructuring of molten salt networks induced by dissolved multivalent metal species; (b) Raman spectra (at 20 °C) after 3 h exposure of the Gd_2_O_3_ (6 mol%) – FLiNaK system at 560,600 °C;^96^ (c) XRD analysis of the quenched sample Gd_2_O_3_ (6 mol%) – FLiNaK.^96^ This figure has been adapted from ref. 96 with permission from Elsevier, copyright 2026.

### 4.2 Dynamic phase reconstruction and regeneration-controlled recovery

While the pathways described above capture the primary reduction mechanisms, they do not fully explain the fate of partially reduced or unreacted rare-earth species remaining in complex slag matrices. These residual oxides, oxyhalides, and fluorides can undergo further transformation under molten-salt conditions and re-enter the electrochemical recovery pathway.^86,87,89–91^ Thus, the unified mechanism can be further extended from a single dissolution–migration–reduction sequence to a dynamically coupled process involving phase reconstruction, dissolution, coordination, and renewed electroreduction. Such transformations are driven by local compositional and chemical-potential differences.^89–91^

At the solid–liquid interface, phase reconstruction can convert refractory rare-earth phases into more chemically accessible halide-containing species, which subsequently undergo coordination with the surrounding molten salt:

16 RE_2_O_3_ + 6X^−^ → 2REX_3_ + 3O^2−^

17 RE^3+^ + *n*X^−^ ⇌ [REX_*n*_]^(3−*n*)−^

The coordinated species can then participate in cathodic reduction:

18 RE^3+^ + 3e^−^ → RE

This regeneration pathway is supported by phase-reconstruction studies showing that refractory rare-earth fluoride and oxyfluoride phases can be transformed into more chemically accessible phases.^89–91^ For example, LaF_3_ and LaO_0.7_F_1.6_ in electrolytic slag were reconstructed into La_10_(SiO_4_)_6_O_3_, demonstrating the continued chemical reactivity of residual rare-earth phases.^90^ Meanwhile, the coexistence of metallic Nd and Pr with residual NdF_3_, PrF_3_, and DyF_3_ after electrolysis indicates that phase transformation and metal deposition can proceed concurrently.^79^ FPMD simulations further showed that increasing NdF_3_ concentration reorganizes the local ionic structure of molten LiF–NdF_3_, with NdF_7/8_ polyhedra governing percolation-limited ion transport and the emergence of Nd–Nd–Nd networks associated with phonon softening and reduced ionic conductivity.^87^ These results demonstrate that melt composition regulates the coordination environment and transport characteristics of Nd-containing species, providing molecular-level support for the coordination and mass-transport components of the proposed mechanism rather than direct evidence for Nd^3+^ reduction to metallic Nd. Complementary electrochemical measurements in LiF–NdF_3_–Nd_2_O_3_ melts at 1323 K reported reduction peak potentials of −0.78 and −1.19 V for Nd(iii)/Nd(ii) and Nd(ii)/Nd, respectively.

Accordingly, rare-earth recovery can be viewed as a cyclic extension of the unified framework:

19 Phase reconstruction → RE reduction

Rather than representing an independent mechanism, phase reconstruction provides an additional route for restoring the chemical accessibility of residual rare-earth species. However, direct *in situ* identification of transient RE-halide species and their regeneration during electrolysis remains limited. The proposed cycle should therefore be regarded as a mechanistically supported framework linking phase evolution, coordination reorganization, and renewed electroreduction, rather than a fully resolved elementary reaction sequence.^76,92–95^

Based on the aforementioned coupled reduction and phase-rearrangement mechanisms, molten salt electrolysis provides a fundamentally distinct strategy for recovering rare earth elements from spent NdFeB magnets, integrating thermodynamic activation, electrochemical reduction, and phase reconstruction within a single reaction environment. Rare-earth recovery can therefore be described as a coordination-controlled and phase-reconstructive electrochemical process, where ionic interactions and interfacial reactions collectively determine macroscopic recovery behavior. This intrinsic coupling enables not only efficient extraction but also the continuous transformation and reuse of intermediate phases, distinguishing molten salt recycling from conventional linear approaches.

The fundamental conversion of rare-earth oxides into halide-coordinated species and their subsequent electrochemical reduction has been consistently demonstrated across various molten salt systems. These well-established processes represent the most reliable mechanistic framework for rare-earth recovery. However, the detailed evolution of coordination structures, transient intermediate species, and multi-ion interactions remains strongly dependent on electrolyte composition and operating conditions, and is often inferred from indirect experimental evidence. Therefore, further advances require *in situ* characterization and theoretical modeling capable of resolving high-temperature coordination environments and electrode–electrolyte interfaces, enabling a transition from empirical optimization toward mechanism-driven design of molten salt recycling systems.

## 5 Mechanisms of nuclear materials and radioactive waste in molten salt systems

Molten salt technologies are attracting increasing attention in nuclear fuel recycling and radioactive waste management due to their chemical and radiation stability at high temperatures. The high radiation tolerance of molten salt systems makes them particularly attractive for the treatment and resource recovery of radioactive nuclear waste and spent nuclear fuels. Under highly radioactive conditions, molten salt electrochemical processes can facilitate the selective separation and recovery of valuable metals, offering a promising route for advanced nuclear fuel recycling.

Applications of molten salt technologies in nuclear waste management can generally be categorized into three major areas: recycling of spent nuclear fuel, recovery of waste cladding materials, and treatment of other radioactive wastes. Although these applications involve different feedstocks and processing conditions, their recovery processes can be understood within the same electrochemical framework, while the dominant reaction pathway varies with the chemical state, stability, and composition of the target materials.

### 5.1 Spent nuclear fuel

Mechanistically, the recycling of spent nuclear fuel is governed by electrochemical–chemical coupling processes, which represent one of the most extensively studied application areas in molten salt electrochemistry. Comprehensive reviews of pyroprocessing indicates that electrochemical reduction and electrorefining in molten chloride salts can efficiently recover actinide elements from oxide fuel, thereby improving nuclear resource utilization and reducing the long-term radiotoxicity of radioactive waste.^97,98^ Furthermore, a report by the International Atomic Energy Agency (IAEA) emphasize that advanced recycling technologies such as molten salt pyroprocessing play a critical role in the future management of spent nuclear fuel and radioactive waste.^99–102^ Among these processes, the electrochemical reduction of oxide fuels constitutes the primary step, as it converts stable oxide phases into electrochemically active metallic forms.

#### 5.1.1 Electrochemical reduction of oxide fuels

Electrolytic reduction is a cornerstone of pyroprocessing, enabling the transformation of uranium oxides into metallic uranium in molten LiCl–Li_2_O. This process exemplifies a typical electrochemical–chemical (EC) coupling mechanism, in which electrochemically generated lithium acts as a transient reductant. Lithium ions are first electrochemically reduced at the cathode:

20 Li^+^ + e^−^ → Li

which subsequently reacts chemically with uranium oxides:

21 U_*x*_O_*y*_ + 2*y*Li → *x*U + *y*Li_2_O

while oxide ions migrate to the anode, releasing oxygen gas, as illustrated schematically in Fig. 5(a):

22 O^2−^→½O_2_ + 2e^−^

**Fig. 5:**
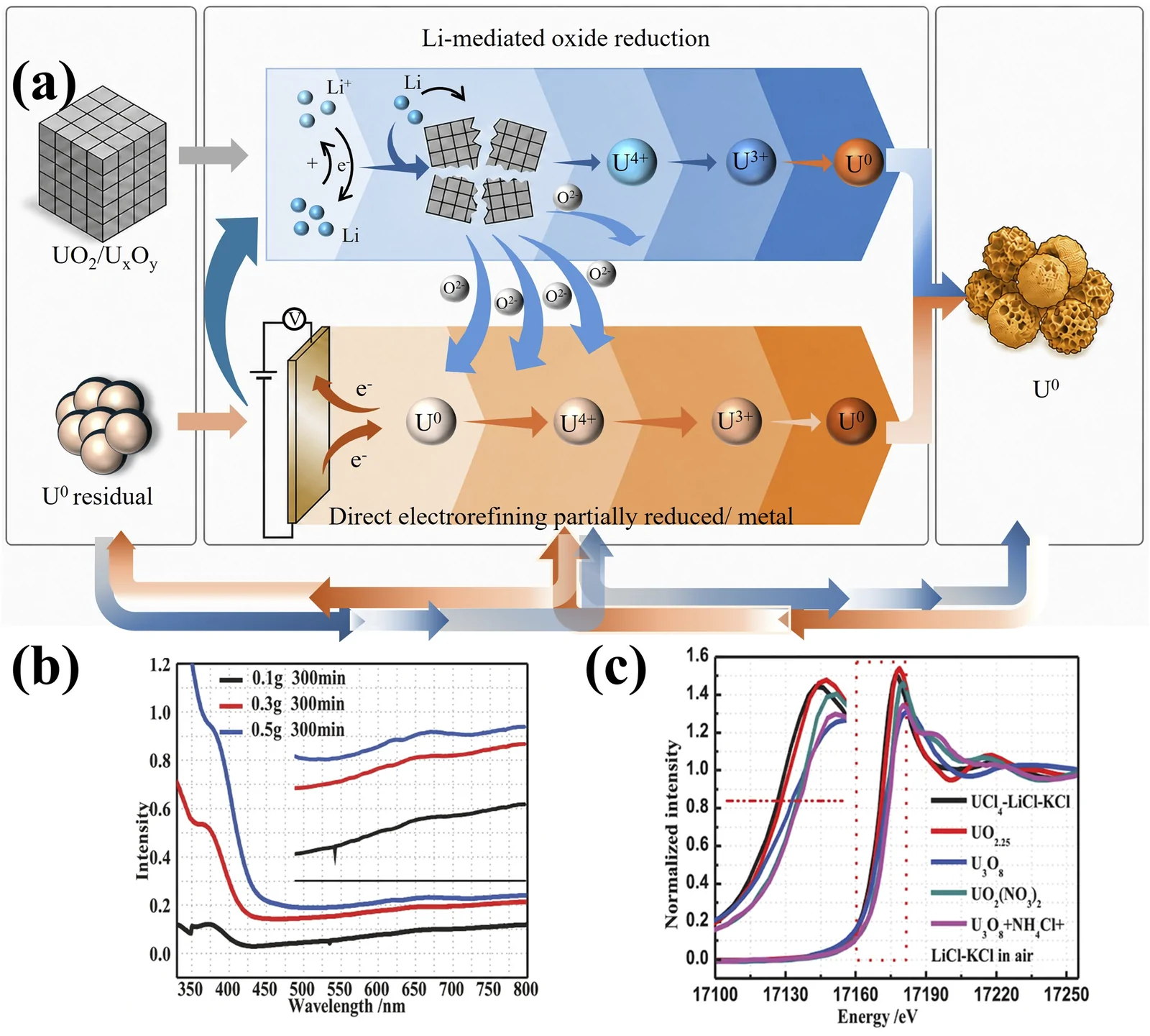
(a) Mechanism of uranium recovery *via* temperature-driven coordination evolution and electrochemical reduction in molten salts; (b) high temperature UV-vis spectra of U_3_O_8_ and NH_4_Cl in LiCl–KCl at 450 °C. Reprinted with permission from ref. 106; (c) U-LIII edge XANES spectra of quenched melt and reference compounds of U in different oxidationstates.^106^ This figure has been adapted from ref. 106 with permission from Elsevier, copyright 2020.

Thus, the key mechanistic feature of spent-fuel recycling is the introduction of a chemical reduction step between electrochemical electron transfer and metal formation. Electrochemical generation of Li provides the reducing species, which subsequently reacts with uranium oxides to remove oxygen from the oxide lattice. The process is therefore governed by the coupling of lithium generation, chemical deoxidation, and oxide-ion transport, while oxygen-ion activity influences oxide stability and oxygen removal.

This lithium-mediated electrochemical–chemical (EC) pathway enables controlled reduction of uranium oxides. Hur *et al.*^103^ demonstrated the metallization of U_3_O_8_ in LiCl–Li_2_O molten salt through a catalytic EC mechanism, in which Li_2_O electrolysis and U_3_O_8_ reduction occurred simultaneously at the cathode. Choi *et al.*^104^ investigated the effect of the cathode-to-anode surface-area ratio on the electrochemical reduction of UO_2_ in molten LiCl–Li_2_O. Bench-scale experiments conducted at 650 °C and a constant cell voltage of 3.2 V examined surface-area ratios ranging from 10.2 to 1.6, revealing that a lower ratio promoted higher current density. Based on these findings, a ratio of 2.6 was adopted in a 17 kg-scale test, in which porous UO_2_ pellets were successfully reduced to metallic uranium. These results highlight the role of electrode geometry in regulating interfacial current density and influencing the scale-up of oxide electroreduction.^101^ Thermodynamic analysis further indicates that the reduction behavior of actinide oxides is governed by oxo-acidity and oxide-ion activity in molten LiCl.^105^ Together, these studies indicate that lithium generation, oxide-ion transport, electrode configuration, and local thermodynamic conditions collectively regulate uranium oxide reduction.

Spectroscopic, electrochemical, and modeling evidence collectively supports the proposed lithium-mediated reduction pathway. Liu *et al.*^106^ found that the U-LIII-edge position was 17 173.40 eV under air, corresponding to an average U oxidation state of +5.98, whereas Ar conditions shifted the edge to 17 171.8–17 172.5 eV with an average valence of +4.72–+5.09. EXAFS further identified U–O and U–Cl shells at 1.77 and 2.67 Å with coordination numbers of approximately 2 and 4, respectively, consistent with [UO_2_Cl_4_]^2−^, while additional U–O (2.34 Å) and U–U (3.89 Å) shells under Ar confirmed the formation of UO_2_. Electrochemical studies further identified lithium uranates as intermediate phases during U_3_O_8_ reduction in LiCl–Li_2_O, with cathodic current efficiencies exceeding 75%. The quantified Li diffusion coefficient of 8.6 × 10^−4^ cm^2^ s^−1^ further indicates that lithium transport within the molten electrolyte is an important kinetic factor controlling the EC reduction pathway.^107^ These results establish that uranium oxide reduction involves atmosphere-dependent valence/coordination reconstruction, lithium–uranium–oxygen intermediates, and transport-coupled subsequent metallization rather than direct one-step reduction of U_3_O_8_ to U.

#### 5.1.2 Electrorefining and electrodeposition of actinides

Once the recovered actinide elements have been converted into metallic form, they must be separated from fission products through electrorefining and electrodeposition. In this stage, the mechanism shifts from chemically assisted oxide reduction toward selective ionic transport and cathodic deposition. Uranium exists primarily as UCl_3_ in the molten salt and can be deposited at the cathode:

23 U^3+^ + 3e^−^ → U

Electrorefining exploits differences in the reduction behavior of actinides and fission products, which are influenced by the activity and coordination environment of ionic species in molten salts. The effective reduction potential depends not only on the intrinsic redox properties of metal ions but also on their thermodynamic activity, which is affected by chloride or mixed-halide coordination. These coordination environments regulate the stability, transport, and reducibility of actinide species, thereby enabling selective electrodeposition.^108–110^ Wu *et al.*^100^ combined UV-vis and XANES spectroscopy to track the oxidation state of uranium during electrodeposition, confirming in real time that U^3+^ is reduced stepwise toU^0^ while remaining stable in the solution. Jung *et al.*^111^ quantitatively compared Bi and Cd liquid-metal electrodes for the electrochemical separation of uranium and lanthanides (Nd, Ce and La) in molten LiCl–KCl at 723.15 K. Under a constant electrodeposition current of −25 mA, approximately 75% of the uranium was deposited from the melt within 1 h in the Bi system, compared with approximately 35% in the Cd system. The corresponding deposition fractions reported for the lanthanides were approximately 45% and 8%, respectively, based on changes in the concentrations of dissolved metal ions. Subsequent dissolution experiments showed that the lanthanides dissolved before uranium from the Bi electrode, whereas uranium and the lanthanides dissolved concurrently from the Cd electrode. These results demonstrate that liquid-metal electrode composition affects deposition kinetics and dissolution selectivity, providing quantitative evidence for interfacial control of actinide–lanthanide separation.^111^

Thermodynamic modeling complements experimental observations. Sridar *et al.*^112^ predicted the phase equilibria and liquidus temperatures in the KCl–LiCl–NaCl–UCl_3_ system, providing thermodynamic guidance for electrorefining conditions. Complementary electrochemical studies have elucidated the dissolution and metal recovery behavior of complex materials; for example, Zhang *et al.*^113^ investigated the electrochemical dissolution of cemented carbide scrap and the subsequent preparation of W and Co metals. Such integration of thermodynamic prediction, electrochemical characterization, and interfacial analysis provides a mechanistic basis for understanding and optimizing selective metal recovery in molten-salt systems.

Beyond actinide recovery efficiency, the long-term sustainability of molten-salt pyroprocessing also depends on the stability of residual salts and the environmental behavior of secondary wastes. Relevant quantitative indicators include radionuclide partitioning between recovered products, residual salts, and solid wastes; radionuclide release and leaching rates under representative disposal conditions; and the chemical stability and residual radioactivity of waste forms during long-term storage. These parameters provide a basis for evaluating radionuclide immobilization, potential environmental release, and the suitability of secondary wastes for subsequent treatment, storage, or disposal. Their systematic assessment is therefore essential for establishing the environmental safety of molten-salt electrochemical recovery processes.

### 5.2 Waste cladding materials

Beyond fuel materials, zirconium-based cladding constitutes another major category of nuclear waste, requiring distinct recycling strategies. In addition to recovering actinides from nuclear fuels, molten salt technologies have also been investigated for recycling zirconium-based cladding materials used in nuclear reactors. Zircaloy cladding hulls represent a major component of solid waste generated during nuclear fuel reprocessing.

Several strategies have been developed for zirconium cladding recycling, as summarized in Fig. 6. These methods are not sequential steps but rather alternative approaches that depend on the processing objectives and the condition of the material.

**Fig. 6:**
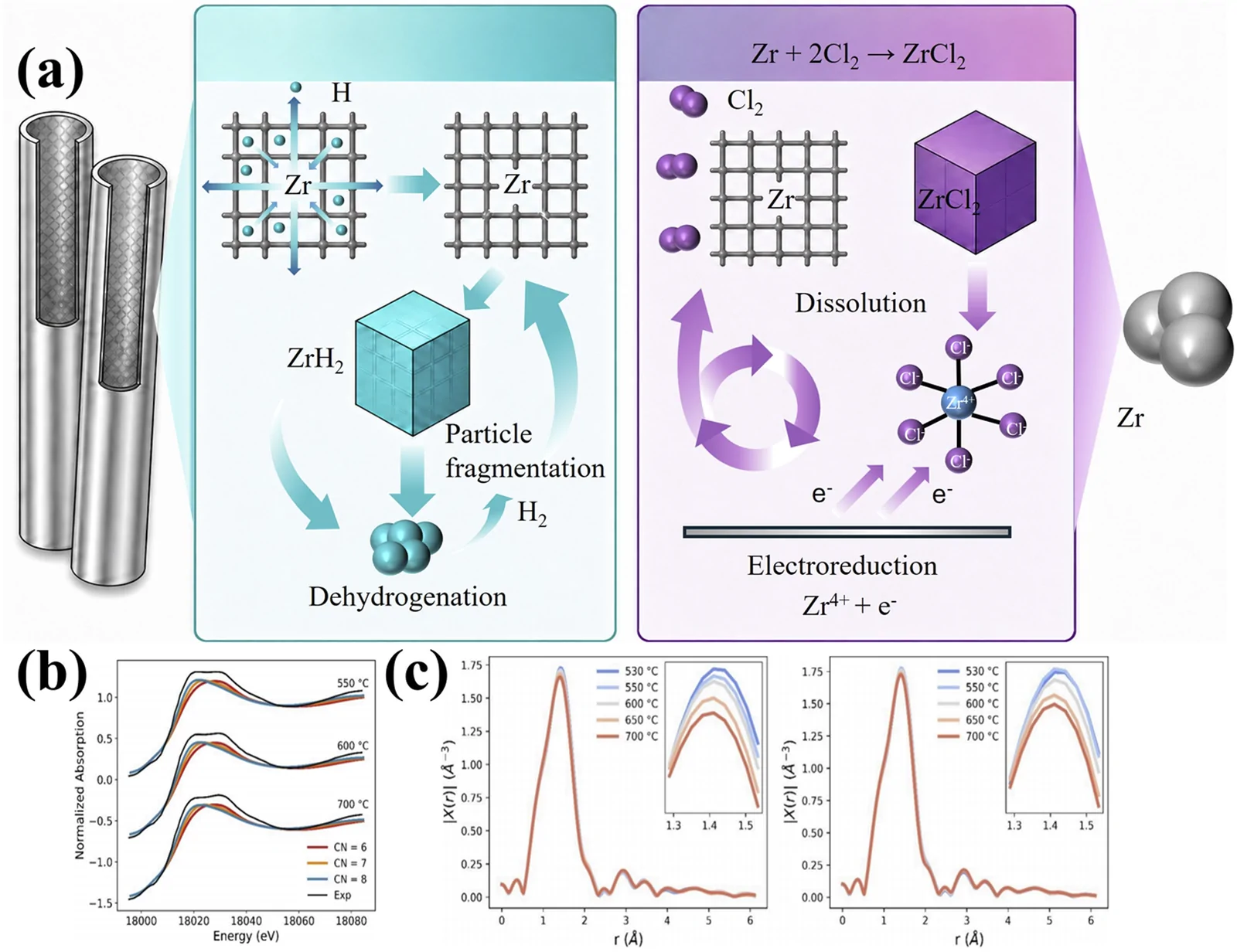
Structure-dependent reactivity of dispersed metal species, highlighting the role of atomic dispersion in molten salt systems.

One promising method for recycling zirconium cladding involves the hydride–milling–dehydride (HMD) process, which converts zirconium alloys into powder through hydrogen absorption followed by thermal decomposition.

24 Zr + H_2_ → ZrH_2_

Subsequently, this brittle zirconium hydride phase can be ground into a fine powder through mechanical grinding. Subsequent heat treatment removes hydrogen and enables the recovery of metallic zirconium:

25 ZrH_2_ → Zr + H_2_

An integrated recycling system based on this process was proposed by Kelley and McDeavitt^114^ to improve zirconium recovery efficiency and reduce the volume of radioactive waste generated by cladding hulls.

Another approach involves the chlorination of zirconium alloys to produce zirconium chlorides.

26 Zr + 2Cl_2_ → ZrCl_4_

Experimental and theoretical studies conducted by Jeon *et al.*^115^ investigated the chlorination reaction behavior of Zircaloy-4 cladding hulls and analyzed the kinetics and thermodynamics of the chlorination process.

Molten salt electrorefining has also been investigated as a method for directly recovering zirconium from cladding hulls. In molten LiCl–KCl electrolytes, zirconium dissolves as zirconium ions and can be electrochemically reduced at the cathode:

27 Zr^4+^ + 4e^−^ → Zr

Electrorefining experiments conducted by Lee *et al.*^116^ demonstrated the feasibility of recovering zirconium from Zircaloy-4 cladding hulls in molten salts. Cyclic voltammetry at 500 °C revealed multiple reduction and oxidation peaks associated with different zirconium oxidation states, while constant-potential deposition at approximately −1.15 V *vs.* Ag/AgCl produced zirconium-containing deposits. The recovered zirconium reached a purity of 99.94 wt%, providing quantitative evidence for selective zirconium recovery.^116^ Complementary electrochemical studies further identified a two-step reduction pathway of Zr(iv) to metallic Zr *via* Zr(ii), with the first reduction step, Zr(iv) + 2e^−^ → Zr(ii), occurring at approximately −1.05 V *vs.* Ag/AgCl (1.0%), followed by the reduction of Zr(ii) to metallic Zr at approximately −1.30 V *vs.* Ag/AgCl (1.0%) at 550 °C. The diffusion coefficients of Zr(iv) were determined to be approximately 4.26 × 10^−5^ cm^2^ s^−1^ by cyclic voltammetry and 4.98 × 10^−5^ cm^2^ s^−1^ by chronopotentiometry.^117^ These quantitative electrochemical results indicate that zirconium speciation, mass transport, and the local electrochemical conditions at the electrode/electrolyte interface collectively govern zirconium reduction and deposition in molten salts. The principal recycling pathways for zirconium cladding hulls are summarized schematically in Fig. 6.

Recent spectroscopic, electrochemical, and computational studies provide complementary evidence for the key transformations involved in zirconium recovery. Raman spectroscopy of Zircaloy-4 detected characteristic signals at 215 and 1187 cm^−1^, which were assigned to δ-ZrH 1.66 and spatially correlated with visible hydride precipitates.^118^ These observations provide direct experimental evidence for hydride formation and support the hydride-related phase transformation involved in zirconium cladding treatment. In molten NaF–ZrF_4_, *in situ* XANES and EXAFS measurements revealed temperature-dependent changes in Zr coordination states and Zr–F bond distances, with increasing temperature driving the melt toward lower Zr coordination numbers and greater local structural distortion.^119^ Complementary *ab initio* and neural-network molecular dynamics simulations reproduced the experimentally observed structural evolution, providing atomic-scale validation of the temperature-dependent coordination reorganization of Zr species in fluoride melts.^119^ These results directly support the coordination step of the proposed molten-salt recovery mechanism.

### 5.3 Other radioactive waste

As a third-generation technology, molten salt technology can also process other radioactive waste streams, providing a versatile platform for the recovery and separation of valuable elements from various radioactive waste streams-particularly uranium-level liquid waste containing actinides and rare earth fission products. A key application lies in the recovery of transuranium elements, which can be recycled for advanced nuclear fuel cycles, thereby contributing to both resource utilization and long-term radiotoxicity reduction.

The fundamental mechanism underlying these processes is governed by the coupling between coordination-controlled dissolution and electrochemical reduction in molten salt media, as illustrated in Fig. 7(a). Rare-earth and actinide species do not exist as free ions, but are stabilized as halide-coordinated complexes, whose local structure determines their thermodynamic stability and electrochemical behavior. Recent studies have demonstrated that lanthanides can exist as atomically dispersed coordination species in molten salts, as illustrated in Fig. 7(b), rather than aggregated ionic phases. XAFS analyses further confirm that these species are surrounded by well-defined coordination environments, which evolve dynamically with melt composition and temperature.^120^

**Fig. 7:**
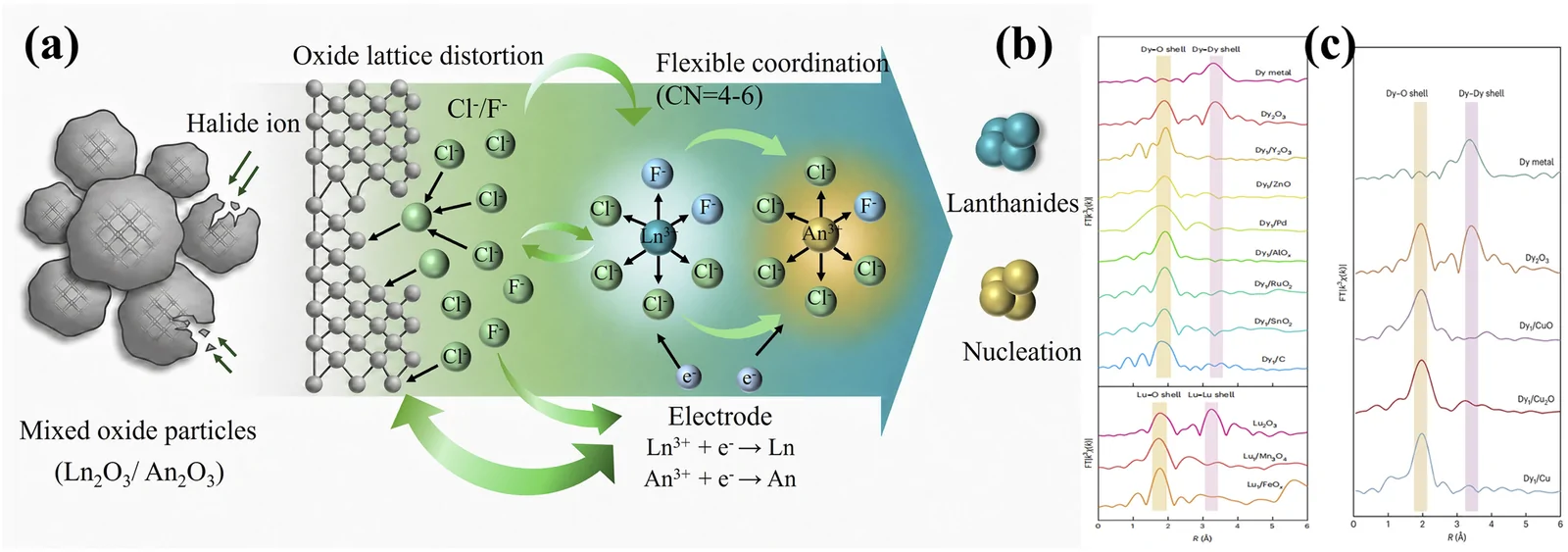
(a) Dynamic structural evolution during electrochemical reduction from coordinated ions to metallic states; (b) dispersing Ln(s) within different substrates, including Dy for U_2_O_3_, ZnO, Pd, AlO_*x*_, RuO_2_, SnO_2_, commercial XC-72 and Lu for Mn_3_O_4_ and FeO_*x*_. Reprinted with permission from ref. 125; (c) EXAFS at the Dyl_3_ edge of Dy_1_/CuO, Dy_1_/Cu_2_O and Dy_1_/Cu. Dy_1_/Cu was measured during an *in situ* cathodic reduction. Dy metal and Dy_2_O_3_ are shown as references. FT, Fourier transform. Reprinted with permission from ref. 125. This figure has been adapted from ref. 125 with permission from Springer Nature, copyright 2026.

The conversion of oxides into electrochemically active species occurs *via* coordination-driven reactions, such as the conversion of rare-earth oxides into chlorides or fluorides, which has been experimentally verified as a prerequisite for electrochemical reduction.^86^ Furthermore, the incorporation of fluoride ions significantly modifies coordination structures, thereby altering reduction potentials and electrochemical pathways.^109^ Rare-earth and actinide species are first stabilized as halide-coordinated complexes, followed by electrochemical reduction *via* well-defined redox pathways.

The study by Park *et al.*^121^ provides a classic example of this mechanism, wherein they systematically investigated the electrolytic reduction behavior of spent oxide fuel in LiCl–Li_2_O molten salts and demonstrated that the process proceeds *via* a solid-state electro-deoxidation pathway, as evidenced by *in situ* EXAFS analysis in Fig. 7(c). In this mechanism, oxygen ions are continuously removed from oxide matrices and transported through the molten salt electrolyte, resulting in the progressive reduction of the oxide phase to metallic uranium. For UO_2_, this electrochemical deoxidation can be represented by

28 UO_2_ + 4e^−^ → U + 2O^2−^

This oxygen-ion-mediated pathway is accompanied by distinct morphological evolution, in which dense oxide phases gradually transform into porous metallic structures, thereby facilitating electrolyte penetration and enhancing reaction kinetics.

In addition to actinide recovery, molten salt systems have also been employed for the separation of rare earth fission products. Wang *et al.*^122^ successfully achieved the electrochemical extraction and separation of praseodymium and erbium using a reactive magnesium electrode in molten salts. On an inert W electrode, the reduction of Pr(iii) and Er(iii) proceeds through one-step three-electron reactions, which can be expressed as:

29 Pr^3+^ + 3e^−^ → Pr

30 Er^3+^ + 3e^−^ → Er

When a reactive Mg electrode is used, the electrochemically generated Pr and Er subsequently react with the Mg substrate to form intermetallic compounds. The experimentally identified products include Mg_3_Pr and Mg_24_Er_5_, while Mg_12_Pr was obtained during the potentiostatic separation of Pr(iii) from a Pr(iii)–Er(iii) mixture.^122^ In this process, magnesium acts as a sacrificial reductant, forming intermetallic compounds or alloys with rare earth elements, thereby enabling their selective removal from the molten salt phase. The separation mechanism depends on the differences in redox potentials among rare earth elements and their alloying behavior, which are directly influenced by their coordination environment in molten salts. Electrochemical studies have shown that lanthanide reduction involves coordinated ionic species, while the nucleation and growth of deposited metals are strongly influenced by the electrode surface and melt structure.^105,121^ In particular, Vandarkuzhali *et al.*^124^ investigated Nd(iii) electrochemistry in LiCl–KCl melts over 723–798 K using cyclic voltammetry, convolution voltammetry, chronopotentiometry, and square-wave voltammetry, identifying the sequential reduction of Nd(iii) to Nd(ii) and subsequently to metallic Nd. At 723 K, the cathodic peak potentials for the Nd(iii)/Nd(ii) and Nd(ii)/Nd reduction steps were approximately −1.85 and −2.05 V, respectively, against an Ag/Ag^+^ reference electrode containing 0.8 wt% AgCl; the corresponding open-circuit equilibrium potentials were approximately −1.842 and −2.015 V, respectively. These quantitative electrochemical results link lanthanide speciation with sequential electron-transfer pathways. In related studies, the cathodic reduction peak potentials for Pr(iii)/Pr and Er(iii)/Er in LiCl–KCl melts at 823 K were approximately −2.08 and −2.01 V, respectively, against an Ag/AgCl reference electrode containing 1.0 wt% AgCl. Furthermore, the positive shifts in the reduction potentials of lanthanide ions observed by Wang *et al.*^122^ on reactive Mg electrodes were attributed to the formation of Mg–lanthanide intermetallic compounds, demonstrating that electrode composition can modify interfacial reduction behavior and facilitate the selective extraction of rare-earth elements.

Electrochemical studies have shown that the reduction process of lanthanide ions occurs *via* coordinated Ln^3+^ species, with nucleation and growth behavior strongly dependent on electrode surface and melt structure.^108,123^ Cyclic voltammetry further reveals unique redox responses associated with different coordination states and electrode materials, confirming the intrinsic link between electrochemical behavior and coordination chemistry.^124^

Collectively, molten salt electrolysis in nuclear systems is governed by coupled coordination-controlled dissolution, electrochemical reduction, and ion transport, where coordinated species evolution influences reaction pathways. This enables direct ionic separation and co-recovery of radioactive elements without pure plutonium isolation, offering advantages in proliferation resistance and closed fuel cycles. Compared with aqueous processes, molten salts provide simpler flows, compact reactors, and higher radiation tolerance, although corrosion, fission product accumulation, and transport limitations remain challenges.

Mechanistically, reaction selectivity and efficiency depend on multivalent ion redox behavior, diffusion, and interfacial deposition. Electrochemical measurements, *in situ* spectroscopy, and simulations have validated fundamental UO_2_/U^3+^/U^0^ transformations and selective deposition behaviors, while complex multicomponent systems involving competitive interfacial reactions and transient coordination structures remain insufficiently understood. Further coupling of advanced characterization and modeling is therefore required to enable reliable scale-up.

## 6 Mechanisms of tungsten and cobalt from cemented carbide scraps in molten salts

Unlike the systems discussed earlier, where recovery is primarily governed by phase transformations or dissolution processes, WC–Co cemented carbides are dominated by strong interfacial constraints arising from their composite structure. The Co binder phase forms a continuous metallic network that rigidly locks WC grains within a dense skeleton. As a result, the initiation of recycling does not involve direct tungsten conversion, but rather the destabilization and migration of the Co phase. Only when this interconnected structure is disrupted can WC access reactive interfaces for further transformation, which fundamentally governs the mechanistic divergence among various recycling routes.^126–130^

Since disrupting the continuous Co binder network is prerequisite for exposing reactive WC interfaces, recycling strategies fundamentally depend on how to overcome this interfacial constraint. Accordingly, current approaches can be categorized into three pathways. Direct recycling relies on physical or metallurgical disruption of the binder phase. For instance, during zinc processing, Zn infiltrates the Co phase and forms intermetallic compounds, triggering volume expansion and lattice distortion that promote interfacial cracking. This transforms the continuous metallic network into a brittle structure, enabling particle liberation.^131^ In hydrometallurgical routes, tungsten is first oxidized to alter its chemical state. At elevated temperatures, WC is converted to WO_3_, which subsequently dissolves in the form of tungstate:

31 2WC + 5O_2_ → 2WO_3_ + 2CO_2_

Coupled oxygen diffusion inward and CO_2_ evolution outward progressively convert the dense structure into a porous one, continuously exposing fresh reactive interfaces. In the presence of oxide additives, oxygen transport across the interface is enhanced, thereby shifting the rate-limiting step from interfacial reaction to bulk diffusion.^132–134^

Despite differences in operational details, these methods share a common feature of decoupling structural breakdown from element recovery. While effective, these methods rely on phase separation followed by reassembly, which inherently increases process complexity and energy consumption. In contrast, the molten salt electrolysis strategy proposed by Xiaoli Xi's research group enables the electrochemical separation of W and Co. Unlike the “disassembly–reassembly” approach, this method uses electrochemical driving forces to directly separate and reassemble elements *via* ionic intermediates, thereby enabling structural disintegration and mass transfer to occur simultaneously within a single system. In NaCl–KCl-based melts, by adjusting the salt composition, it is possible to establish correlations between dissolution behavior, ionic speciation, and deposition processes, thereby enabling stepwise separation and *in situ* reconstruction pathways.^126,135,136^

Mechanistically, described as a coupled sequence of anodic dissolution, coordination reconstruction, and cathodic reduction. WC does not undergo a simple direct reduction pathway, but rather follows the pathway of “anodic dissociation – coordination reconstruction in molten salt-cathodic deposition.” At the anode, Co dissolves preferentially:

32 Co → Co^2+^ + 2e^−^

followed by progressive destabilization of the WC framework and release of tungsten-containing species:

33 WC → W^*n*+^ + C + *n*e^−^

Following anodic release, tungsten species undergo a fundamental transition from solid-state carbide bonding to solvated coordination complexes in the molten salt, thereby redefining their reaction pathway. Electrochemical analysis indicates that their behavior is diffusion-controlled, with anodic current scaling linearly with the square root of scan rate and chronoamperometric responses showing characteristic diffusion decay. This suggests that WC decomposition is governed not only by interfacial reactions but also by mass transport within the molten salt. Thus, the anodic dissolution of the composite electrode and subsequent tungsten transport are closely coupled with the coordination environment of the melt.

In particular, during tungsten reduction, fluorides are typically introduced into chloride-based systems to modulate the coordination environment and improve the reduction behavior. Once dissolved, tungsten does not exist as simple ions but forms coordinated complexes, as evidenced by the RDFs and integral curves in Fig. 8(b) and the coordination-number analysis in Fig. 8(c). Its speciation depends strongly on the melt composition, particularly the [F^−^]/[Cl^−^] ratio. With increasing fluoride content, the coordination environment evolves from chloride-dominated to mixed chloro–fluoro and ultimately fluoride-dominated complexes, as quantitatively reflected in Fig. 8(c).

**Fig. 8:**
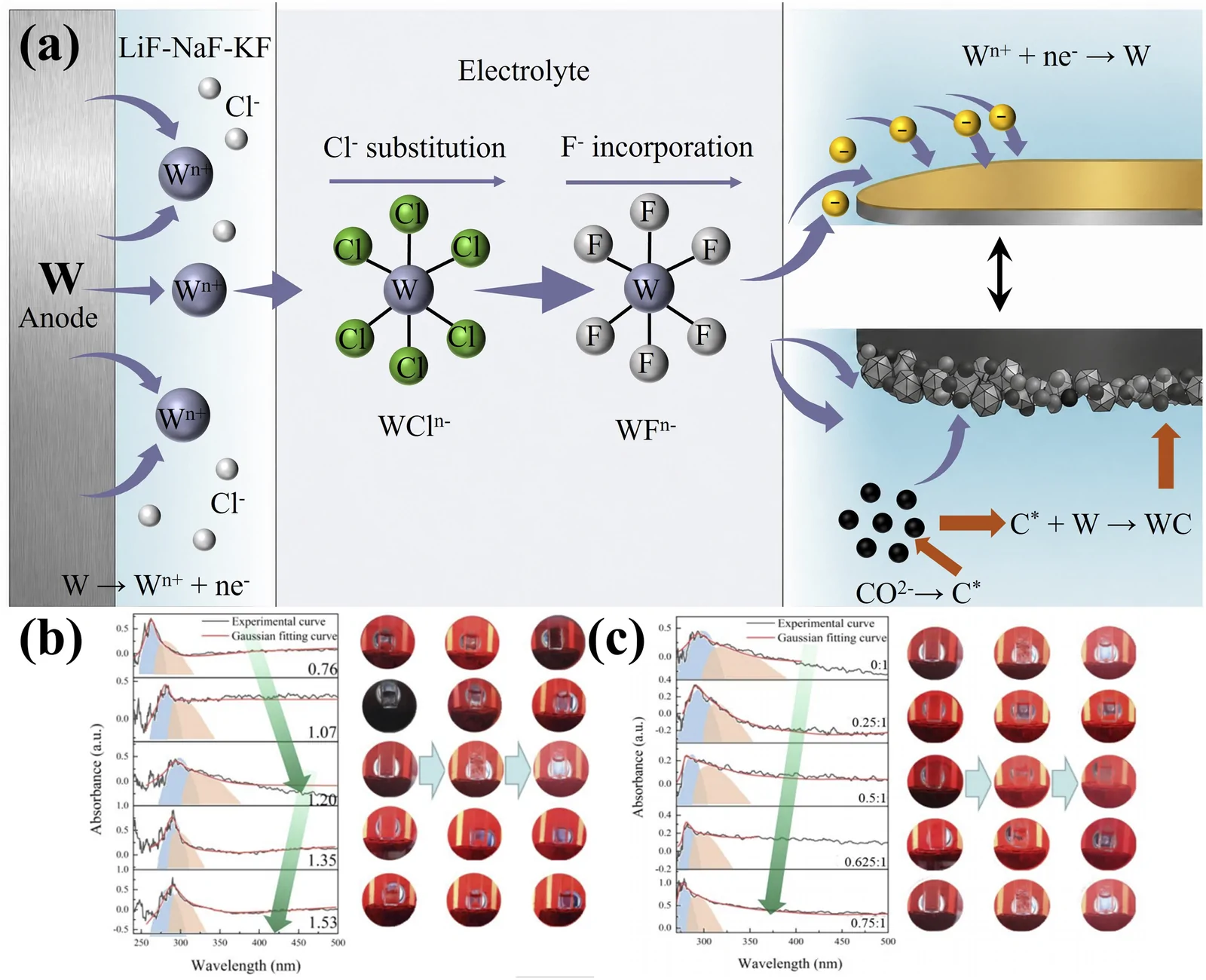
(a) Composition-dependent speciation and reaction pathway modulation of tungsten species in molten salts; (b) RDFs and integral curves of W(vi)–Cl/F and W(ii)–Cl/F after electrolysis of tungsten carbide in the salts with various of [Cl^−^]/[F^−^] ratios;^138^ (c) coordination number of the tungsten ion groups after electrolysis of tungsten carbide in the salts with various of [Cl^−^]/[F^−^] ratios.^138^ This figure has been adapted from ref. 138 with permission from Elsevier, copyright 2022.

This coordination evolution contrasts with the chloride-dominated system shown in Fig. 8(a), where tungsten mainly exists as chloro-coordinated species. With increasing F^−^ content, ligand substitution changes the coordination environment of tungsten and consequently modifies its reduction pathway. Under low [F^−^]/[Cl^−^] ratios, tungsten undergoes a multistep reduction from W(vi) to W(iv) and subsequently W(0), whereas higher [F^−^]/[Cl^−^] ratios favor a simplified pathway involving low-valence intermediates.^127,128^ Electrolyte cations may further affect this process by modifying the coordination configuration and valence-state distribution of tungsten, thereby influencing species transport and interfacial electron transfer.

These proposed relationships are further examined using spectroscopic, electrochemical, and computational approaches. *In situ* high-temperature UV-vis spectra reveal systematic changes in tungsten-related absorption features with varying Cl^−^/F^−^ ratios, providing evidence for the evolution from chloride-to mixed chloro–fluoro-coordinated species and ultimately fluoride-coordinated species.^137^ Differential pulse voltammetry further reveals multiple reduction features under different melt compositions, indicating the involvement of different tungsten valence states during reduction; changes in peak position and intensity with the Cl^−^/F^−^ ratio suggest that coordination changes are accompanied by changes in the accessible reduction states and pathways.^127,128^ DFT calculations further reproduce the experimentally observed spectral characteristics and provide structural information on the corresponding tungsten complexes, thereby linking the observed coordination evolution with tungsten valence-state changes and reduction behavior.^137^

These findings establish that tungsten coordination and valence evolution are closely coupled with its electrochemical reduction behavior. At the cathode, the dissolved metal species are subsequently reduced and deposited:

34 Co^2+^ + 2e^−^ → Co

35 W^*n*+^ + *n*e^−^ → W

Tungsten deposition involves multielectron reduction and may transition from nucleation-to diffusion-controlled behavior. The distinct deposition potentials of W and Co enable their selective separation within appropriate potential windows, providing a basis for stepwise electrolysis and high-purity recovery.^134^

However, tungsten recovery efficiency is constrained by its limited solubility and the restricted dissolution of WC in simple chloride or fluoride melts. The addition of WO_3_ or Na_2_WO_4_ increases the concentration of soluble tungsten species and introduces oxygen-containing species that promote anodic WC dissolution. This enhanced tungsten supply strengthens the coupling between anodic dissolution and cathodic deposition, thereby improving current efficiency.^133,134,139^ In contrast, fluorides primarily regulate tungsten coordination and reduction behavior rather than directly enhancing WC dissolution.

A different strategy is to modify the cathodic process through Na_2_CO_3_ addition. Carbonate provides a carbon source that can participate in cathodic carbon formation, while dissolved W species are simultaneously reduced to metallic W. The co-deposited W and carbon subsequently undergo short-range carbon diffusion, promoting *in situ* carburization and WC formation:

36 CO_3_^2−^ + 4e^−^ → C + 3O^2−^

37 W + C → WC

Thus, oxygen-containing additives primarily enhance anodic WC dissolution and tungsten supply, whereas Na_2_CO_3_ modifies the cathodic deposition process and enables *in situ* carburization. This coupling extends molten salt electrolysis from metal recovery toward direct regeneration of WC-containing materials, offering a potential closed-loop route for cemented-carbide recycling.

Collectively, molten-salt electrolysis of WC–Co is distinguished by the sequential disruption and separation of its binder–carbide composite structure. Preferential anodic dissolution of the Co binder destabilizes the WC framework and exposes tungsten-containing phases to the molten electrolyte, providing a characteristic starting point for electrochemical separation. The subsequent behavior of W is strongly dependent on its coordination environment, with Cl^−^/F^−^ composition regulating tungsten speciation, accessible valence states, and reduction pathways. Thus, the separation of W and Co is governed not simply by their individual electrochemical potentials, but by the interplay between Co binder dissolution and the coordination-controlled reduction of tungsten.

A remaining challenge is to establish how the dissolution of the Co binder influences the subsequent conversion and deposition of tungsten species, particularly under varying Cl^−^/F^−^ and oxygen-containing conditions. Future studies should therefore focus on clarifying this relationship and using it to guide electrolyte composition and electrode-potential design for selective Co removal and controlled W deposition. Such a mechanism-based understanding will be important for improving the controllability and scalability of molten-salt recycling of WC–Co scrap.

## 7 Mechanisms of lithium and cobalt from spent lithium-ion batteries in molten salts

Spent lithium-ion batteries (LIBs) contain Li and transition metals such as Co, Ni, and Mn, making their cathode materials important secondary resources for metal recovery.^140,141^ LiCoO_2_ is a representative cobalt-bearing cathode material that has been widely investigated for resource recovery.^141,142^ In molten salt electrolysis, the recovery of these elements is governed by the structural and electrochemical evolution of the cathode, together with the subsequent dissolution, transport, and partitioning of metal species in the molten electrolyt.^143,144^ Accordingly, this section focuses on two mechanistic processes: the electro-deoxidation and structural transformation of cathode materials, followed by lithium dissolution, partitioning, and recovery in the molten electrolyte. These processes are further coupled with electrochemical oxygen removal and interfacial reduction during molten salt electrolysis, with ion coordination, transport, and cathode structural evolution collectively determining the Li/Co recovery pathways.^145,146^

### 7.1 Electro-deoxidation and structural transformation of cathode materials

In molten salts, cathode oxides undergo stepwise reduction under cathodic polarization. Taking LiCoO_2_ as a representative layered oxide system commonly used in LIB cathodes, oxygen ions are progressively removed from the lattice:

38 LiCoO_2_ → CoO → Co

This sequential electro-deoxidation destabilizes the Co–O framework, triggering lattice reconstruction and the emergence of porous metallic structures. More fundamentally, this structural transformation originates from the instability of the layered LiCoO_2_ framework upon oxygen removal. As oxygen is extracted, the local coordination environment of cobalt is disrupted, thereby promoting transition metal migration and phase transformation toward thermodynamically stable configurations, as supported by first-principles calculations and electronic structure analysis.^147^ Such porosity can facilitate molten salt penetration, increase the accessible reaction interface, and thereby promote further electro-deoxidation. The overall electro-deoxidation and lithium release pathway is schematically depicted in Fig. 9(a). Chronoamperometric and cyclic voltametric studies confirm that the overall reduction is diffusion-limited and primarily depends on the migration of oxygen ions through the partially reduced solid and molten salt.^143,144,148,149^ Accordingly, oxygen-ion transport across the evolving solid/electrolyte interface represents an important kinetic factor in the reduction process. The dominant role of oxygen-ion transport indicates that ionic diffusion and interfacial migration processes largely determine the reduction kinetics. The process exhibits a shrinking-core-type behavior, with the reduction reaction proceeding inward from the particle surface, which is consistent with the results of numerical simulations of oxygen ion transport and interfacial kinetics.^150^ The porous morphology generated by electro-deoxidation not only improves ion and electron transport but also sustains the propagation of the reaction front, consistent with the characteristic behavior of FFC-type processes.^151–154^ These observations indicate that structural evolution and oxygen-ion transport are closely coupled during electro-deoxidation, with the evolving porous layer regulating electrolyte accessibility and the progression of the reduction front.

**Fig. 9:**
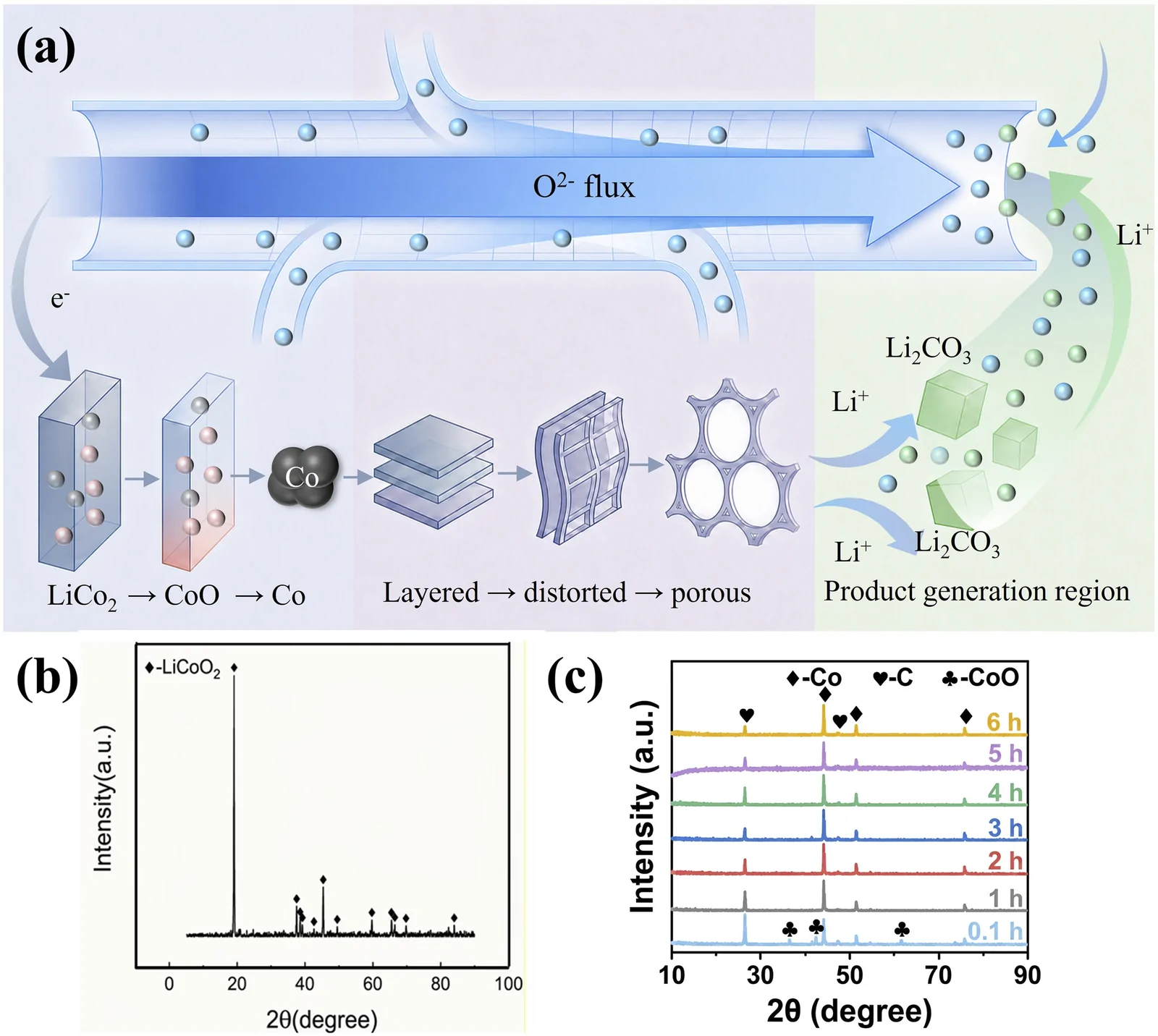
(a) Influence of initial phase structure on dissolution behavior and subsequent electrochemical transformation; (b) XRD patterns of the spend cathode materials. Reprinted with permission from ref. 164. This figure has been adapted from ref. 164 with permission from Elsevier, copyright 2019 (c) and pelleting molar ratios of spent LiCoO_2_ to spend graphite (680 C, 1.5 V, 5 h, 5 MPa). Reprinted with permission from ref. 148. This figure has been adapted from the original source and reproduced in accordance with the applicable open-access license.

Structural and spectroscopic characterizations corroborate these electrochemical observations. XRD and TEM reveal the gradual disappearance of LiCoO_2_ peaks and the formation of CoO and metallic Co phases, as shown in the representative XRD patterns in Fig. 9(b) and (c), while XPS captures the progressive reduction of cobalt from Co^3+^ to Co^2+^ and finally to Co^0^.^148,151^ EXAFS results indicate that local atomic rearrangement occurs alongside framework reconstruction, reflecting the development of metallic coordination. Complementary *in situ* Raman measurements show that the characteristic LiCoO_2_ bands near 485 and 593 cm^−1^ broaden and shift during delithiation, confirming progressive lattice distortion and structural degradation.^151^ Collectively, these electrochemical, structural, and spectroscopic results support a coupled mechanism involving oxygen removal, lattice reconstruction, oxygen-ion transport, and progressive Co reduction.

The anodic reaction provides an additional boundary condition for the oxygen-removal process. Oxygen-containing species transported from the cathode toward the anode undergo anodic oxidation, with the gaseous products depending on the anode material and electrolyte chemistry. Gas evolution may locally disturb the electrode–electrolyte interface and modify electrolyte flow and mass transfer near the anode. Gas bubbles may undergo nucleation, growth, coalescence, and detachment, forming a transient gas–electrolyte boundary layer near the anode. Persistent bubble coverage can reduce the effective anodic area, redistribute the local current density, and increase ohmic losses. Bubble-induced liquid motion may also alter local convection and mass transfer.^46,47^ Potential strategies to mitigate these effects include optimizing anode orientation and surface geometry or employing slotted structures to facilitate bubble detachment and gas release.^48^ Such effects can influence the local transport environment, but they remain secondary to the cathodic electro-deoxidation pathway and do not alter the fundamental sequence of oxygen removal, lattice reconstruction, and Co reduction.

The electrical and thermal characteristics of the electrode–electrolyte system can influence the local reaction environment. In molten-salt electrolysis, the applied current, electrode configuration, and cell geometry affect heat generation and dissipation, making Joule heating an important factor in maintaining suitable thermal conditions.^155^ Conductive carbon materials can also participate in electrothermal processes. For example, CO_2_-derived carbon produced by molten-salt electrolysis can be converted into highly graphitized graphite through subsequent Joule heating at 2800 °C, demonstrating how resistive heating drives structural reorganization and improves the graphitization of carbon materials.^156^ In an adjacent materials system, CNT-assisted liquid-metal composites exhibited an electrical conductivity of 1.0 × 10^6^ S m^−1^, a saturation temperature of 63.0 °C at an applied voltage of 2.0 V, and an out-of-plane thermal conductivity of 2.19 W m^−1^ K^−1^.^157^ These quantitative results illustrate how conductive-network design affects electrical conduction and thermal response. However, they were obtained from flexible polymer-based composites rather than molten-salt electrolysis cells and therefore provide an analogy for electrothermal regulation, not direct evidence of enhanced interfacial charge collection or metal-reduction kinetics. In the present context, electrothermal effects are considered auxiliary factors that may modify local temperature, ionic transport, and interfacial reaction conditions rather than constitute an independent electrochemical reduction pathway.

The electrochemical driving force is further regulated by the applied potential, which determines the thermodynamic feasibility and kinetic accessibility of the reduction process. Overall, the electro-deoxidation of LiCoO_2_ proceeds through a coupled sequence of oxygen removal, oxygen-ion transport, lattice reconstruction, and Co^3+^ → Co^2+^ → Co^0^ reduction, while anodic gas evolution and electrothermal effects primarily act as secondary factors that modulate the local mass-transfer and thermal environment. Beyond electrochemical conversion, downstream separation remains important for selectively recovering individual transition metals from spent cathode-derived streams.^155,158^

### 7.2 Lithium dissolution, partitioning, and recovery

In molten salt systems, the electrochemical behavior of lithium differs markedly from that of transition metals. This distinction originates from its relatively weak Li–O bonding and high chemical activity, which favor its extraction into the molten salt phase during oxygen removal.

During electro-deoxidation, Li is released from the cathode lattice as Li^+^ and transferred into the molten electrolyte, while lattice oxygen is removed through the electrochemical reduction pathway. Thus, lithium recovery involves lattice deintercalation, Li^+^ transfer to the salt phase, and subsequent stabilization and transport within the molten electrolyte rather than direct cathodic deposition.

This reaction pathway indicates that lithium is released through lattice decomposition rather than direct electrochemical reduction. Consequently, lithium is intrinsically decoupled from transition metals and preferentially transferred into the molten salt phase, laying the foundation for its selective separation.^143,149^

Therefore, the preferential dissolution of lithium is not merely a kinetic phenomenon but rather a thermodynamically driven partitioning process. Once released, lithium does not exist as free ions but is stabilized through coordination with surrounding anions in the molten electrolyte, forming dynamic solvation structures that govern its transport and distribution.^159^ The local physicochemical environment of the molten electrolyte can further influence the transport of Li-containing species. In addition to coordination structure and temperature, electrostatic interactions and ionic correlations may contribute to ionic mobility. A data-driven analysis of 218 ionic liquids reported conductivity activation energies in the approximate range of 20–30 kJ mol^−1^ and identified a negative correlation between dielectric permittivity and activation energy in a subset of the systems examined.^160^ This finding suggests that electrostatic screening may be relevant to interpreting ion-transport barriers, although the relationship has not been directly established for high-temperature molten salts. Studies of Li-salt-containing ionic liquids have also highlighted the relationship between Li^+^ coordination, diffusion, and conductivity.^161^ Taken together, these findings support considering electrostatic interactions as a complementary factor when interpreting Li^+^ transport and partitioning. Nevertheless, coordination-mediated species formation, ionic migration, and interfacial electrochemical reactions remain central to the mechanistic interpretation of lithium recovery. First-principles molecular dynamics simulations of LiCl–KCl melts quantified the self-diffusion coefficients of Li and K species at different temperatures and melt compositions, demonstrating that Li-containing species possess composition- and temperature-dependent mobility in the molten electrolyte and thereby providing theoretical support for Li^+^ transport after its release from the oxide lattice.^162^ Due to its high solubility and stability in molten salts, lithium accumulates in the electrolyte, enabling subsequent recovery *via* precipitation reactions. Li *et al.*^143^ demonstrated that dissolved lithium can subsequently be recovered through precipitation reactions with carbonate or phosphate anions:

39 2Li^+^ + CO_3_^2−^ → Li_2_CO_3_↓

or

40 3Li^+^ + PO_4_^3−^ → Li_3_PO_4_↓

as illustrated in the recovery pathway of Fig. 9(a).

The proposed mechanism is further supported by quantitative recovery results and complementary spectroscopic and electrochemical evidence. Feng *et al.*^148^ achieved recovery efficiencies of 99.3% for Li, 98.1% for Co, and 83.6% for graphite at 680 °C and 1.5 V for 5 h, demonstrating pronounced electrochemical separation between Li and Co during molten-salt electrolysis. Cyclic voltammetry further revealed distinct reduction features associated with LiCoO_2_ and cobalt oxide, occurring at approximately −0.9 and −1.3 V (*vs.* Ag/Ag^+^), respectively, confirming the electrochemical differentiation between lithium extraction and transition-metal reduction.^148^ In addition, molecular dynamics calculations have quantified lithium transport behavior in molten chloride systems, showing high Li^+^ mobility and a low migration barrier (∼5.28 kcal mol^−1^) that facilitate lithium partitioning into the molten electrolyte. Mirza *et al.*^149^ further showed that Co is preferentially reduced at the cathode, whereas Li predominantly remains in the molten-salt phase, providing direct evidence for the electrochemical decoupling of Li from the transition-metal component. Salagre *et al.*^151^ used Raman spectroscopy and XPS to show that characteristic Raman bands of LiCoO_2_ at approximately 485 and 593 cm^−1^ broadened and shifted during delithiation, accompanied by changes in the Li/Co signals, indicating lattice reconstruction and structural degradation upon Li extraction. Collectively, these results demonstrate that selective Li recovery is not simply a cathodic reduction process, but proceeds through LiCoO_2_ lattice deintercalation, Li^+^ transfer into the molten salt, salt-phase stabilization and transport, followed by precipitation-based recovery, whereas Co is preferentially recovered through cathodic reduction.

Collectively, these results demonstrate that selective Li recovery is not simply a cathodic reduction process, but proceeds through LiCoO_2_ lattice deintercalation, Li^+^ transfer into the molten salt, salt-phase stabilization and transport, followed by precipitation-based recovery, whereas Co is preferentially recovered through cathodic reduction.

Beyond the mechanistic aspects of Li/Co separation, electrode-interface regulation offers an additional perspective for improving electrochemical processes. Zhang *et al.*^163^ reported that a self-supporting FeCoNi medium-entropy alloy with a nanosheet-like surface, prepared *via* molten-salt electrolysis, exhibited an overpotential of 233 mV at 10 mA cm^−2^ and a Tafel slope of approximately 29.8 mV dec^−1^ for the oxygen evolution reaction, together with stable activity over 500 h. Although this study focused on oxygen evolution rather than metal recovery, it demonstrates how electrode composition and surface morphology can influence interfacial electrocatalytic performance, providing a methodological reference for future electrode-interface design in electrochemical resource recovery.

Overall, molten salt electrolysis provides a distinctive route for spent lithium-ion battery recycling by directly coupling cathode deoxidation with the separation of lithium and transition metals. Rather than requiring complete dissolution of the cathode, electrochemical oxygen removal destabilizes the LiCoO_2_ lattice and promotes the reduction of Co toward the metallic state, while Li is released from the lattice and preferentially transferred into the molten electrolyte. This *in situ* partitioning of Co and Li allows structural transformation, metal reduction, and lithium extraction to occur within a common electrochemical environment, providing a mechanistic basis for selective resource recovery. The key advantage of this approach therefore lies not simply in replacing conventional leaching or thermal treatment, but in using the electrochemical and physicochemical properties of molten salts to control the distinct pathways of Li and transition metals. Further clarification of Li^+^ coordination, transport, and partitioning will be essential for designing electrolytes that enhance this intrinsic separation and improve the efficiency and selectivity of molten-salt recycling of spent LIB cathodes.

## 8 Sustainability implications of molten-salt electrolysis

### 8.1 Circular economy, resource efficiency, and environmental impacts

In the preceding sections, the fundamental mechanisms and recovery characteristics of molten-salt electrolysis have been discussed. From a broader perspective, the sustainability assessment of this technology should consider not only process performance but also resource efficiency, environmental impacts, and technical feasibility within a circular economy framework.

The circular economy concept emphasizes closed-loop resource utilization,^165^ while increasing concerns regarding critical and high-value metals supply have highlighted the importance of secondary resource recovery.^50^ In this context, the environmental performance of metal recovery processes is strongly influenced by system boundaries, energy assumptions, and evaluation methods. Therefore, life cycle assessment (LCA) and techno-economic analysis (TEA) are necessary tools for systematically evaluating the sustainability of molten-salt electrolysis.

Although molten-salt electrolysis can reduce dependence on chemical reagents and secondary waste generation through electrochemical conversion of metal-containing secondary resources, its environmental performance remains strongly dependent on specific process conditions. Since electricity is the primary energy input, factors including electricity source, current efficiency, electrolyte consumption, and recycling rate significantly affect overall environmental impacts. Previous studies have shown that the environmental performance of metallurgical processes is sensitive to energy structure and system assumptions.^165,166^ Similarly, studies on aluminum decarbonization pathways have demonstrated that electricity carbon intensity and recycled material utilization are important factors affecting emission reduction potential.^28^ Therefore, the sustainability advantages of molten-salt electrolysis should be evaluated under consistent system boundaries rather than through generalized comparisons.

A qualitative comparison of different metal recovery routes from the perspective of resource efficiency, energy consumption, chemical requirements, and environmental impacts is summarized in Table 1.

**Table 1:** Qualitative comparison of environmental performance of different metal recovery routes and key influencing factors

Aspect	Pyrometallurgy	Hydrometallurgy	Molten-salt electrolysis
Operating temperature	High	Low-moderate	High
Main energy source	Thermal energy	Chemicals and electricity	Electricity
Chemical consumption	Low	High	Moderate
Wastewater generation	Low	High	Low
Direct CO_2_ emissions	High	Moderate	Potentially low^a^
Metal selectivity	Moderate	High	High
Reagent/electrolyte recyclability	Limited	Limited	Potentially high
Overall environmental performance	Moderate	Moderate	Potentially favorable^a^

a Dependent on electricity mix, current efficiency and electrolyte recycling.

Due to the diversity of secondary resources and differences in feedstock composition, process conditions, and functional units, establishing a unified quantitative evaluation framework remains challenging. Therefore, representative cases with relatively consistent assessment conditions are required for meaningful comparison. In this section, rare-earth recovery from NdFeB magnets is selected as a representative case, and the corresponding environmental, technical, and economic indicators are summarized in Table 2.

**Table 2:** Comparative environmental, technical, and economic performance of NdFeB rare earth recovery *via* molten-salt electrolysis and room-temperature ionic liquid (RTIL) electrochemical processes^77,78,81,170,171^

Indicator	Primary production	Molten-salt electrolysis	Ionic liquid route
Energy demand	High	9–13 kWh kg^−1^ REM	∼3.3 kWh kg^−1^ Nd
CO_2_ emissions (GWP)	9–16 kg CO_2_ eq. per kg Nd	5 kg CO_2_ eq. per kg Nd	Not sufficiently quantified
Recovery efficiency (%)	—	90–95% yield	>99% REE recovery
Chemical consumption	Conventional chemical separation	Fluoride- or chloride-based molten salts	Regenerable/reusable ionic liquids
Economic performance	Established but energy-intensive	High-temperature operation; promising for direct metal production	Low-temperature operation; IL cost and scale-up remain challenges

Despite its potential for secondary resource recovery, molten-salt electrolysis still faces several technical and economic challenges. High-temperature operation requires stable reactor materials and electrolyte management to address issues such as volatilization, compositional changes, and electrode corrosion. In addition, impurities in complex waste streams may influence dissolution, migration, and co-deposition behaviors, affecting current efficiency and product quality. Although the reaction mechanisms have been investigated at laboratory scale, their applicability under continuous and large-scale operation requires further validation. Therefore, integrated evaluation combining LCA, TEA, and process-level performance assessment is necessary to determine the practical sustainability of molten-salt electrolysis for sustainable metal recovery.

### 8.2 Emerging technologies and future directions

Building on the dissolution–migration–reduction and coordination-regulated reduction frameworks established above, recent developments in molten-salt electrolysis have focused on improving individual stages of these pathways. Because MSE performance varies with the metal species, feedstock, electrolyte composition, and electrode configuration, quantitative comparison of representative systems provides a useful basis for evaluating process performance. As summarized in Table 3, reported values of operating temperature, current efficiency, faradaic efficiency, product purity, and energy consumption vary substantially among different metal-recovery systems. However, these parameters are not consistently reported, which limits direct comparison and highlights the need for more standardized performance evaluation.

**Table 3:** Quantitative comparison of representative molten-salt electrolysis processes for metal recovery from secondary resources^11,20,77,78,113,133,139,148,149,167–169^

Metal/resource	Electrolyte	*T*	Electrolysis conditions	Recovery/deposition	Product quality/outcome	Key quantitative evidence
Al scrap	AlCl_3_–NaCl	170	100 mA cm^−2^, 4 h	—	99.7% Al	3–9 kWh kg^−1^ Al
Al–Si waste alloy	AlCl_3_–NaCl–KCl	170	30 mA cm^−2^, 1.5 h	—	99.3% Al	Low-T Al refining
Al scrap	AlCl_3_–NaCl–LiCl–KCl	100	30 mA cm^−2^, 3 h	—	≈99.4 wt% Al	Al_2_Cl_7_^−^-mediated deposition
NdFeB/Nd	LiCl–KCl–NdCl_3_	—	NdCl_3_ 1.65 M	—	>97 wt% Nd	2.3 kWh kg^−1^ Nd; 5 kg CO_2_-eq.
NdFeB scrap	LiF-based fluoride	≈950	15 A lab electrolysis	90–95% material yield^a^	—	9–13 kWh kg^−1^ REM^a^
Nd	LiCl–KCl–AlCl_3_	600	Potentiostatic, 30 h	99.25%	Al_2_Nd/Al_3_Nd	Alloy extraction
Nd	LiCl–KCl + Ga–Al	—	Galvanostatic	—	Nd–Ga–Al alloy	High-efficiency liquid-metal cathode
U–6Zr, unirradiated	LiCl–KCl–UCl_3_	500	Electrorefining	61% U deposited	Zr ≈ 3 mg g^−1^ U	Metallic-fuel recycling
U–6Zr, irradiated	LiCl–KCl–UCl_3_	500	Electrorefining	36% U deposited	Zr ≈ 3 mg g^−1^ U	Hot-cell demonstration
WC–Co	NaF–KF	800	60 mA, 4 h	Selective W recovery	W powder ≈ 500 nm	Strong W selectivity
WC–6Co	NaCl–KCl–WO_3_	750	0.1 A cm^−2^, 8 h	W/Co separation	Single-phase W	WO_3_ promotes anode dissolution
WC–6Co	NaCl–KCl	750	Sacrificial anode	W/Co separation	Pure W + pure Co by XRD/elemental analysis	Multiple-anode recovery demonstrated
WC–23Co	Na_2_WO_4_-6 wt% Na_2_CO_3_	775	Liquid-Zn cathodic protection	WC–Co regeneration	WC + Co composite powder	6 wt% Na_2_CO_3_; ∼450 °C lower than redox route
LiCoO_2_/spent LIB	LiCl–KCl	450	CoO reduction at −2.4 V	—	Co deposition	Fluidized cathode
Spent LiCoO_2_	Molten carbonate	—	Controlled potential	Li ≤ 85%; Co ≤ 99%	CoO/Co + Li_2_CO_3_	Regeneration of LiCoO_2_ demonstrated
Spent LiCoO_2_ + graphite	NaCl–Na_2_CO_3_	680	1.5 V, 5 h	Li 99.3%; Co 98.1%; graphite 83.6%	Co + graphite + Li_2_CO_3_	Simultaneous cathode/anode recovery

a REM denotes rare earth metals. Material yield refers to the recovery yield of rare earth metals. Energy consumption is expressed per kilogram of recovered rare earth metals.

Emerging technologies have therefore been developed mainly to improve specific stages of MSE rather than to replace the overall recovery pathway.^2,17,79^ At the coordination and transport stage, electrolyte regulation can modify the coordination environment of metal ions and consequently influence their electrochemical behavior.^79^ First-principles molecular dynamics further provides insight into the local structure and dynamic behavior of molten salts, supporting mechanistic interpretation of ion transport and electrochemical reactions.^24^

At the reduction and metal-collection stage, liquid-metal cathodes and fluidized cathodes provide alternative configurations for controlling metal deposition and collection. Liquid-metal electrodes based on Bi and Cd have been used for selective separation of uranium and lanthanides, while fluidized cathode systems have been demonstrated for cobalt recovery from spent lithium-ion batteries and tungsten oxide reduction.^111,149,153^ Chemical regulation can also enhance separation; for example, electrochemical alloying coupled with boride formation has been used to regulate the distribution of alkaline- and rare-earth elements.^94^ In addition, kinetic analysis provides quantitative understanding of cathodic reduction and its dependence on operating conditions.^17^

Overall, these developments represent targeted improvements within the established mechanistic framework: coordination and electrolyte regulation influence ion transport, advanced cathode configurations improve reduction and collection, and chemical alloying strategies enhance selective separation.^2,17,79,94,111,150,154^ This step-specific regulation demonstrates how mechanistic understanding can be translated into improved selectivity and efficiency for MSE-based recovery from complex secondary resources.^2,17^

## 9 Conclusions and prospect

Collectively, this review establishes a mechanism-oriented framework for understanding molten salt electrolysis (MSE) of secondary resources. By examining aluminum-containing residues, NdFeB magnets, nuclear materials, cemented carbide scrap, and spent lithium-ion battery cathodes, we show that metal recovery generally involves a sequence of feedstock activation or dissolution, coordination and speciation, ion transport, and interfacial electrochemical reduction, while the dominant step varies with feedstock chemistry and molten-salt composition. The review further summarizes how electrochemical measurements, spectroscopic characterization, and computational modeling can be combined to identify reaction intermediates, ion coordination, transport behavior, and reduction pathways.

Despite these advances, the mechanistic understanding of MSE remains incomplete. Many studies still emphasize recovery efficiency and product characterization, whereas quantitative links between molten-salt speciation, ion transport, interfacial kinetics, and product formation are less systematically established. This limitation is particularly important for complex secondary resources, where multiple metal species and solid phases may undergo simultaneous dissolution, redistribution, and reduction. Under the proposed mechanistic framework, future studies should therefore move beyond empirical optimization of temperature, voltage, and salt composition toward mechanism-based control of electrolyte chemistry, electrode interfaces, and mass transport.

Future work should place greater emphasis on combining *in situ* or *operando* characterization with electrochemical analysis and computational modeling to resolve transient species and reaction pathways. At the same time, mechanistic insights need to be connected with practical metrics, including energy consumption, metal recovery and purity, environmental impacts, and techno-economic performance. Such integration would help identify which mechanistic strategies are transferable across different secondary resources and which are specific to particular feedstock-electrolyte systems, providing a clearer basis for the rational development and scale-up of MSE-based resource recovery.

## Author contributions

Shuaixing Wang: writing – original draft preparation; Ming Feng: writing – review and editing; Jieyang Sun: data curation; Binyao Zhang: validation; Yanfen Li: supervision. All authors have read and agreed to the published version of the manuscript.

## Conflicts of interest

The authors declare no conflicts of interest.

## Data Availability

No primary research results, software or code have been included and no new data were generated or analysed as part of this review.
